# *TRAF7* mutations stabilize K/NRAS and hyperactivate MAPK signaling to cause CAFDADD neurodevelopmental defects

**DOI:** 10.1126/sciadv.adw8672

**Published:** 2026-09-25

**Authors:** Man Xiao, Yang Liu, Xiaozhen Song, Jingxuan Liu, Xiaojun Tang, Maosen Dou, Wei Yu, Hong Zhang, Wenhao Weng, Dingding Han, Xiaoping Lan, Tingting Zhao, Chenji Wang, Lixiang Ma, Nan-Jie Xu, Shengnan Wu

**Affiliations:** ^1^ Department of Clinical Laboratory, Shanghai Children’s Hospital, School of Medicine, Shanghai Jiao Tong University, Shanghai, China.; ^2^ Institute of Pediatric Infection, Immunity, and Critical Care Medicine, Shanghai Jiao Tong University School of Medicine, Shanghai, China.; ^3^ College of Health Science and Technology, Shanghai Jiao Tong University School of Medicine, Shanghai, China.; ^4^ Department of Anatomy and Histology and Embryology, School of Basic Medical Science, Fudan University, Shanghai, China.; ^5^ State Key Laboratory of Genetics and Development of Complex Phenotypes, School of Life Sciences and Zhongshan Hospital, Fudan University, Shanghai, China.; ^6^ Department of Respiratory and Critical Care Medicine, Shanghai General Hospital, Shanghai Jiao Tong University School of Medicine, Shanghai, China.; ^7^ Shanghai Engineering Research Center for Big Data in Pediatric Precision Medicine, Center for Biomedical Informatics, School of Medicine, Shanghai Jiao Tong University, Shanghai, China.; ^8^ Shanghai Pudong Hospital, Fudan University Pudong Medical Center, State Key Laboratory of Genetics and Development of Complex Phenotypes, MOE Engineering Research Center of Gene Technology, Shanghai Engineering Research Center of Industrial Microorganisms, School of Life Sciences, Fudan University, Shanghai, China.; ^9^ Collaborative Innovation Center for Brain Science, Department of Anatomy and Physiology, Shanghai Jiao Tong University School of Medicine, Shanghai, China.; ^10^ NHC Key Laboratory of Medical Embryogenesis and Developmental Molecular Biology and Shanghai Key Laboratory of Embryo and Reproduction Engineering, Shanghai, China.

## Abstract

The MAPK-ERK1/2 pathway plays a crucial role in neurodevelopment during embryogenesis, and the hyperactivation of this signaling cascade serves as a pathological hallmark for various neurodevelopmental disorders. Germline variants in *TRAF7*, encoding a RING-type E3 ubiquitin ligase, are genetically associated with cardiac, facial, and digital anomalies with developmental delay (CAFDADD) syndrome; however, the downstream substrates of TRAF7 and the precise mechanism driving neurodevelopmental defects remain enigmatic. Here, we established a *Traf7*^R654Q/+^ knock-in mouse model and patient-specific induced pluripotent stem cell (iPSC)–derived human cortical organoids (hCOs) carrying the recurrent *TRAF7*^R655Q^ mutation. Heterozygous *Traf7*^R654Q/+^ mice exhibited remarkable growth retardation, skeletal abnormalities, and neurobehavioral deficits, whereas mutant hCOs displayed early cortical neurogenesis defects, characterized by premature neural differentiation and a depleted progenitor pool. Mechanistically, biochemical analyses revealed that TRAF7 interacts with KRAS and NRAS to promote their polyubiquitination and subsequent proteasomal degradation, maintaining physiological homeostatic control over the downstream MAPK cascade. CAFDADD-associated TRAF7 variants exert a dominant-negative effect, disrupting WT protein function and causing the posttranslational accumulation of KRAS and NRAS, which fundamentally drives constitutive MAPK-ERK1/2 pathway hyperactivation. Notably, pharmacological inhibition of MEK1/2 with selumetinib suppressed ERK1/2 hyperactivation, partially mitigated aberrant neuroepithelial morphology and neural differentiation in *TRAF7*^R655Q/+^ hCOs, and improved brain-to-body weight ratios in *Traf7*^R654Q/+^ mice. Together, our findings identify TRAF7 as a critical posttranslational regulator of RAS proteostasis during development and uncover a pivotal mechanistic link between impaired RAS ubiquitination and CAFDADD pathogenesis, providing preliminary clinical-front evidence for targeting the MAPK pathway to manage ongoing developmental deficits in TRAF7-associated disorders.

## INTRODUCTION

Cardiac, facial, and digital anomalies with developmental delay (CAFDADD) syndrome is a recently identified congenital developmental disorder characterized by multisystemic clinical manifestations (OMIM: 618164) (*1*–*4*). Heterozygous germline mutations in tumor necrosis factor receptor–associated factor 7 (*TRAF7*), which encodes a RING-type E3 ubiquitin ligase, have recently emerged as the primary causative factors for this syndrome. To date, virtually all identified CAFDADD-associated *TRAF7* variants are recurrent missense mutations, strongly suggesting that disease pathogenesis is driven by specific alterations in protein function, such as dominant-negative effects, rather than simple haploinsufficiency. Consistent with this notion, the high tolerance of *TRAF7* to loss-of-function variants within the gnomAD database (pLI = 0.02) further argues against haploinsufficiency as the primary driver underlying this severe pediatric disease (*2*, *4*). Notably, CAFDADD syndrome exhibits considerable phenotypic heterogeneity, reflecting the inherently complex and context-dependent nature of *TRAF7*-associated pathogenic mechanisms. Although substantial clinical overlap exists across affected individuals, remarkable phenotypic variabilities are consistently observed both among individuals carrying the same variants and across different *TRAF7* variants. Affected individuals typically present with varying degrees of recognizable dysmorphic facial features (such as epicanthal folds, ptosis, and low-set or malformed ears), skeletal defects, extremity abnormalities (including digit deviations and aberrant palmar creases), congenital cardiac anomalies, motor disabilities, and intellectual impairments. Additionally, a subset of patients displays autism spectrum disorder (ASD)–like social challenges, language delays, and mild anxiety. In ongoing clinical practice, specific therapeutic interventions targeting *TRAF7* mutation–driven CAFDADD remain severely limited, with current management strategies being predominantly supportive.

TRAF7 belongs to TRAF family with seven members and is involved in multiple intracellular signaling pathways and multiple biological processes including ubiquitination and myogenesis (*5*). The protein structure comprises an N-terminal RING finger (RING) domain, an adjacent TRAF-type zinc finger domain, a centrally situated coiled-coil (CC) motif, and a unique C-terminal seven WD40 repeats (WDR) domain (*5*). Synergy between TRAF7 and MEKK3, with its WDR domain interacting with MEKK3, activates c-Jun N-terminal kinases JNK1/2, p38 mitogen-activated protein kinases (MAPKs), and the nuclear factor κB pathway (*6*). Homozygous *Traf7*^−/−^ knock-out (KO) disrupts embryonic endothelial integrity, conferring heart defects and embryonic lethality in utero around midgestation through dysfunction in the *Mekk3*-*Mek5*-*Erk5* signaling axis (*7*). In parallel, Traf7 knockdown in *Xenopus* and zebrafish causes embryonic developmental defects, including cardiac, craniofacial, and ciliary abnormalities (*8*, *9*). Studies conducted with fibroblasts derived from patients with CAFDADD show that *TRAF7* variants tend to enhance cell viability, and their transcriptomic analysis reveals numerous differentially expressed genes (DEGs) involved in cardiovascular, skeletal, and nervous system development (*2*). However, the physiological substrates and functions regulated by germline *TRAF7* mutations, as well as the pathogenic mechanisms underlying CAFDADD, are still poorly understood.

The RAS-MAPK pathway represents a fundamental signaling cascade essential for normal development, orchestrating cellular processes including cell proliferation, differentiation, survival, and motility (*10*). The activation of MAPK cascades involves sequential phosphorylation events in the RAS–RAF–MAPK kinase (MEK)–extracellular signal–regulated kinase (ERK) kinase pathway (*11*). Germline alterations in the key components of this pathway affect MAPK-ERK1/2 activation, leading to Noonan-spectrum disorders (NSDs), a group of autosomal dominant genetic malformation disorders (*12*, *13*). NSDs encompass several distinct yet related conditions: Noonan syndrome (NS) caused by mutations in *PTPN11*, *KRAS*, *NRAS*, *RAF1*, and *SOS1* (*13*); Costello syndrome associated with mutations in *HRAS* (*14*); and cardiofaciocutaneous syndrome linked to mutations in *MAP2K1/2* and *BRAF* (*15*) among others, all of which are also increasingly classified as RASopathies (*12*, *13*). These disorders share overlapping clinical features, including craniofacial abnormalities, musculoskeletal defects, ocular anomalies, developmental delay, congenital heart defects (CHDs), intellectual disability, and cancer predisposition (*12*, *13*). Additionally, MAPK-ERK1/2 signaling dysregulation has also been implicated in ASD, Fragile X syndrome, and attention-deficit hyperactivity disorder (*16*, *17*). All of these observations underscore the critical roles of the MAPK-ERK1/2 pathway in both normal embryonic and postnatal development.

We have successfully established induced pluripotent stem cells (iPSCs) from a Chinese female patient with CAFDADD carrying the *TRAF7*^R655Q^ mutation and her healthy father (*18*). In the present study, we generated a corresponding *Traf7*^R654Q/+^ knock-in (KI) mouse model and *TRAF7*^R655Q/+^ human cortical organoids (hCOs) derived from iPSCs of our patient with CAFDADD, providing experimental systems for investigating CAFDADD pathogenesis and its underlying molecular mechanisms. Using these *TRAF7*-mutated models, we demonstrate that ubiquitin-mediated dysregulation of the K/NRAS-MAPK pathway contributes to CAFDADD-associated defects, thereby offering mechanistic insight into *TRAF7*-associated disease and a rationale for exploring target therapeutic strategies.

## RESULTS

### Heterozygous *Traf7*^R654Q/+^ KI mice exhibit remarkable growth retardation and multiorgan abnormalities

We previously reported a patient with a recurrent variant, c.1964G>A (p.R655Q) in *TRAF7*, whose clinical features substantially overlapped with those seen in patients with identified CAFDADD (*18*). The R655Q is a unique hotspot mutation located in the WDR domain of TRAF7, occurring in ∼31.3% (30 of 96) of patients with CAFDADD. To better investigate the role of the *TRAF7* mutations associated with CAFDADD, a KI C57BL/6N mouse model harboring the *Traf7*^R654Q^ substitution (equivalent to the R655Q variant identified in patients with CAFDADD) was generated using embryonic stem (ES) cell targeting technology (fig. S1A). The successful introduction of the *Traf7*^R654Q^ mutation was confirmed by offspring genotyping (fig. S1B) and Sanger sequencing (fig. S1C). Among the 40 blastocysts derived at embryonic day 3.5 (E3.5) by in vitro fertilization, we identified four homozygous *Traf7*^R654Q/R654Q^ embryos (fig. S1D). Notably, no homozygous offspring were recovered at postnatal day 9 (P9) among a cohort of 186 mice generated from heterozygous *Traf7*^R654Q/+^ intercrosses (fig. S1E). This significant deviation from Mendelian inheritance patterns suggests embryonic lethality of biallelic KI mutations, similar to the survival profile of *Traf7*^−/−^ mice (*7*). Further temporal expression analysis demonstrated that *Traf7* mRNA was highly expressed during embryogenesis, with a marked decline postnatally (fig. S1F). These results highlight the critical role of *TRAF7* during embryonic development and imply that biallelic *TRAF7* missense mutations likely lead to embryonic lethality. This observation is consistent with the fact that all reported *TRAF7* mutations associated with CAFDADD have been identified as heterozygous.

**Fig. 1.**
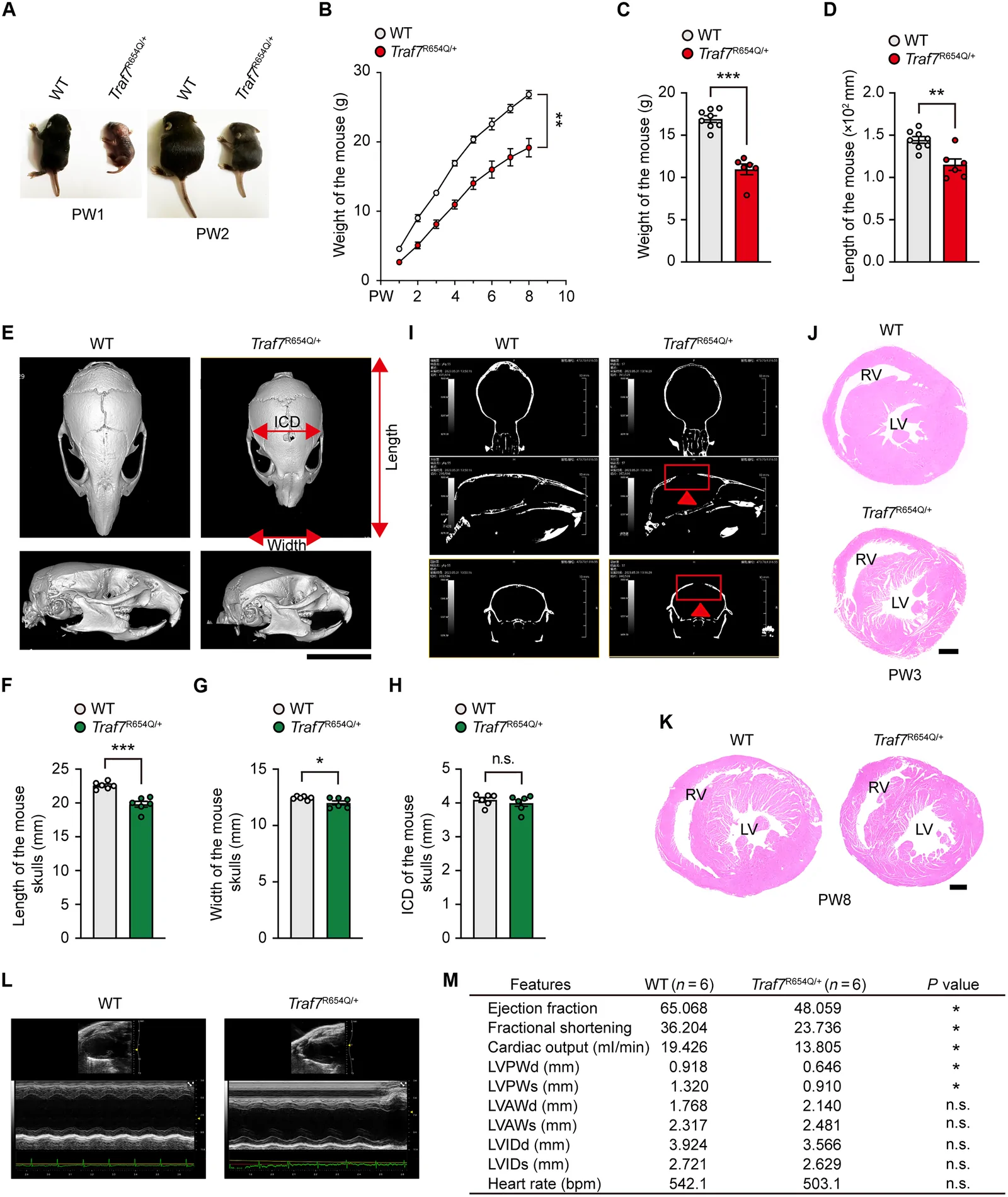
Heterozygous *Traf7*^R654Q/+^ KI mice exhibit remarkable growth retardation and multiorgan abnormalities. (**A**) Representative images of *Traf7*^R654Q/+^ and wild-type (WT) mice at PW1 and PW2. Left: WT; right: *Traf7*^R654Q/+^. (**B** to **D**) Morphometric values are plotted as a growth curve (B) of *Traf7*^R654Q/+^ (*n* = 6) and WT (*n* = 8) mice from PW1 to PW8. The weight (C) and length (D) of *Traf7*^R654Q/+^ and WT mice at PW4. (**E** to **H**) Representative micro-x-ray computed tomography imaging of *Traf7*^R654Q/+^ and WT mouse (*n* = 6) skulls is shown in (E). Scale bar, 10 mm. The indicated values show the average measurements (in millimeters) for the length (F), width (G), and intercanthal distance (ICD; H) of *Traf7*^R654Q/+^ and WT mice. (**I**) Two-dimensional (2D) morphology of the skulls from the coronal, sagittal, and axial planes. Patent cranial sutures are indicated by red rectangles and arrows. (**J** and **K**) Hematoxylin and eosin staining of cross-sectional cardiac sections from *Traf7*^R654Q/+^ and WT mice at PW3 (J) and PW8 (K). Heart chambers: RV, right ventricle; LV, left ventricle. Scale bars, 500 μm. (**L**) Echocardiography imaging of the longitudinal cardiac plane from *Traf7*^R654Q/+^ and WT mice (*n* = 6) at PW8. (**M**) Summary of longitudinal cardiac echocardiography data from *Traf7*^R654Q/+^ and WT mice (*n* = 6) at PW8. Cardiac function parameters: ejection fraction (EF), fractional shortening (FS), cardiac output, left ventricular posterior wall thickness at end-diastole/systole (LVPWd/s), left ventricular anterior wall thickness at end-diastole/systole (LVAWd/s), left ventricular internal dimension at end-diastole/systole (LVIDd/s), and heart rate. Data are presented as means *±* SEM. Two-way analysis of variance (ANOVA) tests were used in (B). Unpaired two-tailed Student’s *t* tests or Mann-Whitney *U* tests were used as appropriate in (C), (D), (F), (G), (H), and (M). *P < 0.05, **P < 0.01, ***P < 0.001; n.s., not significant.

Given that growth and developmental delay are commonly observed in patients with CAFDADD (*1*, *2*), we first assessed growth and development in our mouse model. At postnatal week 1 (PW1) and PW2, *Traf7*^R654Q/+^ mice were visibly smaller than their wild-type (WT) littermates (Fig. 1A). This size discrepancy persisted, with significantly reduced body weight and length observed from PW1 to PW8 (Fig. 1, B to D), indicating that *Traf7*^R654Q/+^ mice exhibit remarkable growth retardation. Further analysis revealed significantly reduced brain, heart, and kidney weight-to-body weight ratios in *Traf7*^R654Q/+^ mice compared with those in WT littermates at PW3 (fig. S2, A and B), PW8 (fig. S2, C and D), and PW15 (fig. S2, E and F). In contrast, lung and liver ratios remained unchanged. *Traf7*^R654Q/+^ mice displayed age-dependent spleen enlargement, with smaller spleen at PW3 transitioning to splenomegaly at PW8 and PW15 (fig. S2, A to F). This dynamic pattern suggests that *TRAF7* mutations may impair spleen development and postnatal extramedullary hematopoiesis. However, spleen abnormalities have been observed in only two patients with CAFDADD and the *TRAF7*^K346E^ and *TRAF7*^T601A^ mutations (*1*).

Because facial dysmorphia, skeletal defects, and extremity and ocular abnormalities were frequently observed in patients with CAFDADD, we further investigated whether these abnormalities were recapitulated in *Traf7*^R654Q/+^ mice. The length, width, and intercanthal distance (ICD) of skulls were measured using micro-x-ray computed tomography. *Traf7*^R654Q/+^ mice displayed statistically significant skull defects, including reduced length and width compared with WT littermates, and no statistical changes in ICD were observed (Fig. 1, E to H). Additionally, *Traf7*^R654Q/+^ mice showed patent cranial sutures and limb defects at PW8 (Fig. 1I and fig. S3A), suggesting that *TRAF7* mutations may impair osteoblast differentiation, further leading to skeletal abnormalities. Notably, *Traf7*^R654Q/+^ mice developed age-related ocular manifestations, including ptosis, exophthalmos, and corneal changes (whitening and cloudiness) at PW28, PW36, and PW38 (fig. S3, B to D). Among 162 *Traf7*^R654Q/+^ mice over 8 weeks old, we found that 124 exhibited ocular manifestations only, 6 exhibited limb defects only, 2 exhibited both ocular and limb defects, and 30 exhibited normal eyes and limbs (fig. S3E). Notably, most of these ocular abnormalities were observed in *Traf7*^R654Q/+^ mice older than 20 weeks, but not in younger mice. These late-onset phenotypes in mice may reflect a form of premature aging, while this differs from the congenital palpebral fissure anomalies consistently reported in patients with CAFDADD.

Cardiac abnormalities are common and highly variable in patients with CAFDADD, including patent ductus arteriosus (PDA), septal defects, and valvular defects (*1*–*4*). We therefore performed a series of cardiac assessments in *Traf7*^R654Q/+^ mice. Histological analysis revealed a substantial thinning of the left ventricle (LV) wall in *Traf7*^R654Q/+^ mice at PW3 (Fig. 1J) and PW8 (Fig. 1K), consistent with a hypoplastic left ventricle (HLV). Echocardiographic evaluation further confirmed impaired cardiac function, with significantly reduced ejection fraction (EF), fractional shortening (FS), cardiac output, and left ventricular posterior wall thickness at end-diastole/systole (LVPWd/s) in *Traf7*^R654Q/+^ mice compared with those in WT littermates (Fig. 1, L and M, and fig. S4, A to G). Together, these findings suggest that severe myocardial dysplasia may contribute to impaired cardiac function. Although heart rate changes were not statistically significant, some individuals displayed mild bradycardia (Fig. 1M and fig. S4G), which may further contribute to cardiac dysfunction. Overall, these data suggest that myocardial dysplasia underlies the cardiac defects and impaired systolic function observed in *Traf7*^R654Q/+^ mice. However, the consistent HLV phenotype exhibited by this mouse model at both PW3 and PW8 differs substantially from the predominant cardiac spectrum observed in patients with CAFDADD (*1*). Clinical data indicate that PDA is the most common cardiac anomaly in patients, followed by septal and valvular defects (*4*). These findings highlight the notable species-specific variations and indicate that this single-mutant model does not fully capture the clinical cardiac spectrum of CAFDADD syndrome. Collectively, *Traf7*^R654Q/+^ mice exhibit developmental delays and multiorgan abnormalities, including craniofacial dysmorphism, skeletal defects, and cardiac abnormalities.

### *Traf7*^R654Q/+^ mice show social deficits, cognitive impairment, and motor abnormalities

**Fig. 2.**
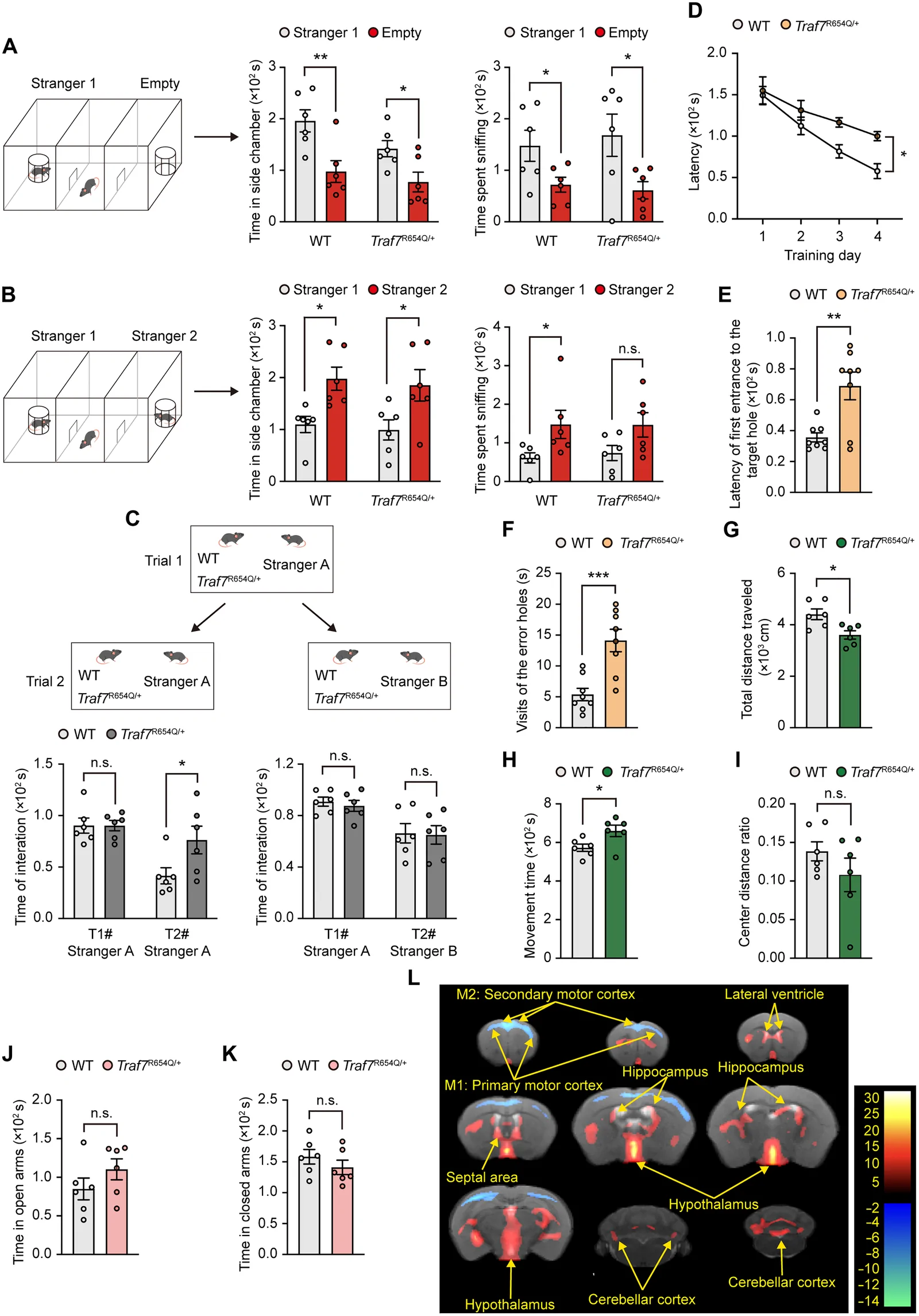
*Traf7*^R654Q/+^ mice show social deficits, cognitive impairment, and motor abnormalities. (**A** and **B**) Social behavior of *Traf7*^R654Q/+^ and WT mice (*n* = 6) at PW8 is measured by three-chamber paradigms. Time in side chambers and time spent sniffing of *Traf7*^R654Q/+^ and WT mice are shown in the right panels. (A) Stranger 1–empty trial, representing social interaction. (B) Stranger 1–stranger 2 trial, representing social novelty preference. (**C**) Direct social interaction test using either a familiar or an unfamiliar stimulus mouse across two trials (top). Interaction times of *Traf7*^R654Q/+^ and WT mice (*n* = 6) during trial 1 and trial 2 when exposed to the familiar or unfamiliar stimulus mice are measured (bottom). (**D**) Spatial learning performance in the Barnes maze test (*n* = 8 per genotype) is measured by the latency to reach the target hole across training days. (**E** and **F**) Latency to find the target hole (E) and the number of visits to the error holes (F) on day 5. (**G** and **H**) Locomotor ability in the OF test is assessed by the total distance traveled (G) and movement time (H) for *Traf7*^R654Q/+^ and WT mice (*n* = 6). (**I** to **K**) Anxiety-like behavior is evaluated by the center distance ratio [movement distance in the center/total distance traveled in open field (OF)] (I) and the time spent in the open (J) or closed (K) arms of the EPM test (*n* = 6 per genotype). (**L**) Voxel-based morphometry analysis of T2-weighted sMRI reveals significant differences in GMVs between *Traf7*^R654Q/+^ and WT mice (*P* < 0.05, clusters > 200 voxels, uncorrected, followed by FDR correction with significance of 0.05). Coronal sections of mouse brain are overlaid on an averaged sMRI template. Data are presented as means *±* SEM. Two-way ANOVA tests were used in (A) to (D). Unpaired two-tailed Student’s *t* tests were used in (E) to (K). *P < 0.05, **P < 0.01, ***P < 0.001; n.s., not significant.

Patients with CAFDADD often display learning/memory deficits and ASD-like social challenges (*2*). To evaluate whether the *Traf7*^R654Q^ mutation affects social behavior and partner discrimination, we used a three-chamber social arena to evaluate social interaction and social novelty preference. *Traf7*^R654Q/+^ mice and WT littermates at PW8 were first tested in a partner-empty trial, which included a partner (stranger 1) in one chamber and an empty cage in another. These results were validated through both chamber time and sniffing time measurements. Both groups displayed comparable social interaction with stranger 1, indicating preserved basic sociability in *Traf7*^R654Q/+^ mice (Fig. 2A). However, in a subsequent test introducing an unfamiliar partner (stranger 2) into the previous empty cage, *Traf7*^R654Q/+^ mice showed similar sniffing times with stranger 1 and stranger 2, suggesting impaired social recognition memory and social novelty preference (Fig. 2B).

To further assess the partner identification and discrimination capabilities, a direct social interaction test using two-step sequential behavioral trials was conducted. These trials included an unfamiliar-familiar trial (stranger A–stranger A) or unfamiliar-unfamiliar trial (stranger A–stranger B), specifically probing short-term social memory (*19*). When reexposed to the familiar partner, WT littermates exhibited the expected reduction in interaction time, whereas *Traf7*^R654Q/+^ mice maintained consistent interaction levels. Conversely, both groups displayed stable interaction patterns when encountering the unfamiliar strangers (Fig. 2C). These findings suggest that the familiar partner discrimination deficits in *Traf7*^R654Q/+^ mice likely stem from impaired social memory.

To explore whether the *TRAF7* mutations affect motor ability or emotional state, a series of behavioral tests were conducted to evaluate exploratory and learning/memory performance. The Barnes maze test was used to assess spatial learning and memory by evaluating a mouse’s ability to find the location of a target hole using visual cues. *Traf7*^R654Q/+^ mice exhibited impaired spatial learning ability, requiring a significantly longer latency to locate the target hole during training days 1 to 4 compared with WT littermates (Fig. 2D). In the probe trials on day 5, *Traf7*^R654Q/+^ mice exhibited spatial memory defects, taking more time and making more errors in locating the target hole (Fig. 2, E and F). Motor function was assessed using the open-field (OF) test, where *Traf7*^R654Q/+^ mice demonstrated reduced locomotor activity, as evidenced by decreased total distance traveled and movement time (Fig. 2, G and H). However, no anxiety-like behavior was observed in *Traf7*^R654Q/+^ mice, as assessed by the center distance ratio in the OF test (Fig. 2I) and the time spent in the open versus closed arms in the elevated plus maze (EPM) test (Fig. 2, J and K).

Clinical neuroimaging of patients with CAFDADD frequently reveals structural abnormalities, such as hydrocephalus, abnormally shaped cerebellum, prominent ventricles, and cerebral atrophy (*1*, *2*). To investigate potential neuroanatomical abnormalities in *Traf7*^R654Q/+^ mice, in vivo structural magnetic resonance imaging (sMRI) was performed. Voxel-based morphometry analysis revealed significant differences in gray matter volumes (GMVs) between *Traf7*^R654Q/+^ mice and WT littermates. Notable increases in GMVs were observed in regions such as the cerebellar cortex (CBC), hippocampus (HP), hypothalamus (HT), lateral ventricle (LVE), and septal area of *Traf7*^R654Q/+^ mice (Fig. 2L). However, the motor cortex exhibited a significant reduction in GMVs, correlating with the observed motor deficits in behavioral tests. These results strongly demonstrate that the *Traf7*^R654Q^ mutation in mice leads to intrinsic impairments in social recognition, cognitive performance, and motor abilities, rather than deficits secondary to heightened anxiety, highlighting the critical role of TRAF7 in neurodevelopment and cognitive function.

### The hyperactivation of the MAPK-ERK1/2 pathway in *Traf7*^R654Q/+^ mice

**Fig. 3.**
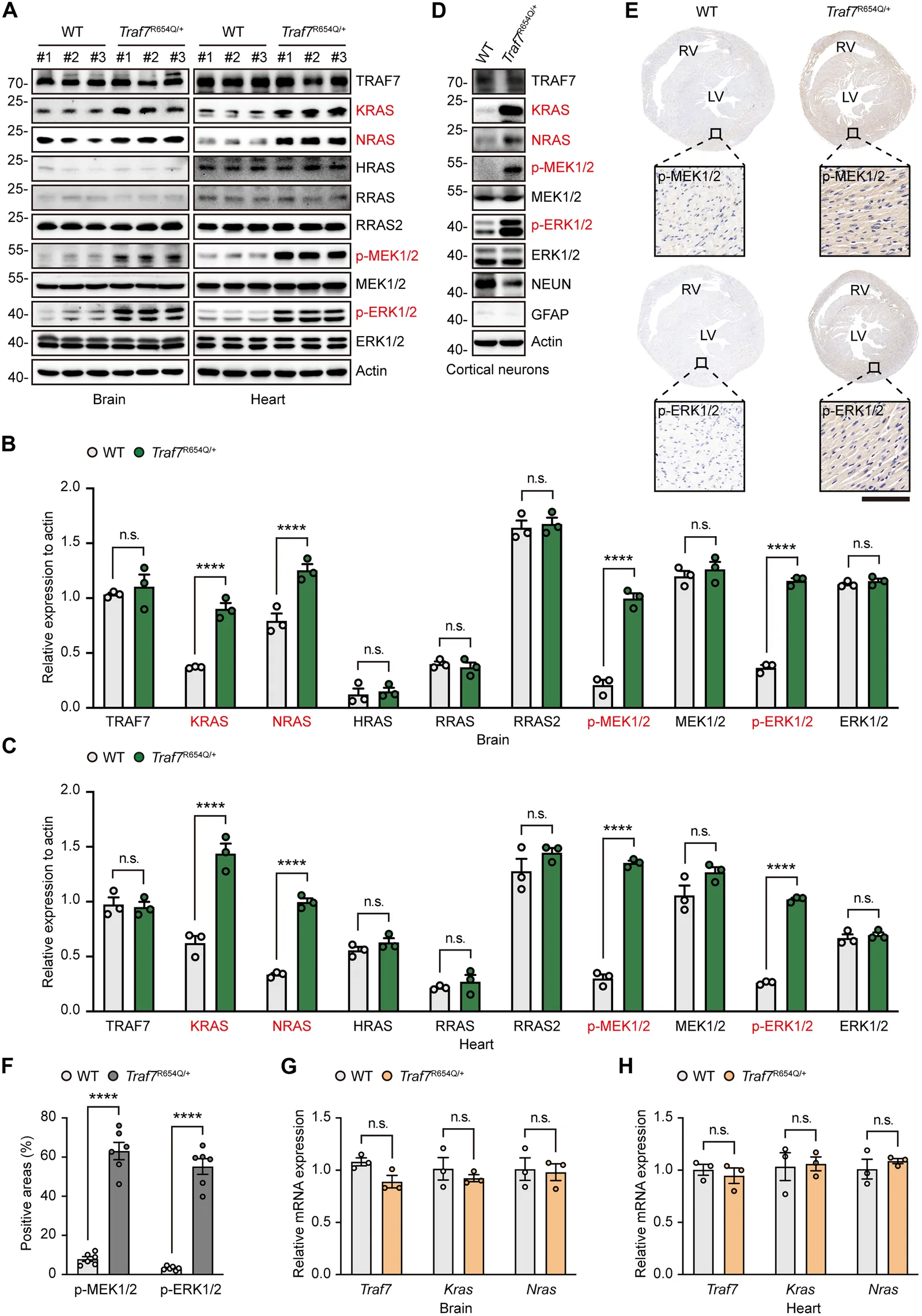
The hyperactivation of the MAPK-ERK1/2 pathway in *Traf7*^R654Q/+^ mice. (**A**) WB analysis of indicated proteins in whole-cell lysates (WCLs) from brain and heart tissues of *Traf7*^R654Q/+^ and WT mice. (**B** and **C**) Quantification of the intensity of indicated proteins from (A), which is normalized to the intensity of actin. (**D**) WB analysis of indicated proteins in WCLs from *Traf7*^R654Q/+^ and WT mice PCNs. (**E** and **F**) Immunohistochemistry analysis of p-MEK1/2 and p-ERK1/2 in cardiac sections from *Traf7*^R654Q/+^ and WT mice (*n* = 6) at PW3. Scale bar, 100 μm. The relative intensity of indicated proteins is quantified and shown in (F). (**G** and **H**) Reverse transcription quantitative PCR (RT-qPCR) measurement of *Traf7*, *Kras*, and *Nras* mRNA expression in brain (G) or heart (H) tissues of *Traf7*^R654Q/+^ and WT mice. Data are presented as means *±* SEM. Two-way ANOVA tests were used in (B), (C), (F), (G), and (H). ****P < 0.0001; n.s., not significant.

The clinical manifestations of patients with CAFDADD partially overlap with those of neurodevelopmental syndromes driven by aberrant MAPK signaling, a pathway that is critical for neurogenesis (*1*). In line with this, *Traf7*^R654Q/+^ mice display phenotypic abnormalities similar to those reported in mouse models with altered ERK1/2 activity (*16*, *20*). These findings collectively prompted us to examine whether MAPK-ERK1/2 signaling is dysregulated by *TRAF7* mutations in vivo*.* At first, we collected brain and heart tissues from P0 mice. Western blot (WB) analysis showed that total levels of MEK1/2 and ERK1/2 were not affected, but phosphorylation levels of MEK1/2 and ERK1/2 were markedly up-regulated in brain and heart tissues from *Traf7*^R654Q/+^ mice compared with those from WT littermates (Fig. 3, A to C). Given the cortical abnormalities observed in sMRI of *Traf7*^R654Q/+^ mice, prepared primary cortical neurons (PCNs) from P0 mice were isolated and cultured in vitro. Consistently, the phosphorylation levels of MEK1/2 and ERK1/2 were markedly up-regulated in *Traf7*^R654Q/+^ PCNs compared with those in WT PCNs (Fig. 3D). Immunohistochemistry analysis also demonstrated that the phosphorylation levels of MEK1/2 and ERK1/2 were markedly elevated in heart tissues from *Traf7*^R654Q/+^ mice compared with those from WT littermates (Fig. 3, E and F).

To investigate the molecular mechanism underlying *TRAF7* mutation–driven MAPK-ERK1/2 hyperactivation, we first detected the level of MEKK3, a TRAF7 binding partner known to activate ERK1/2, JNK, and p38, in the brain and heart tissues of *Traf7*^R654Q/+^ mice. WB analysis revealed that neither MEKK3 levels nor MEKK3-mediated activation of JNK or p38 were affected (fig. S5A). Additionally, a recent study showed that TRAF7 interacted with and controlled the ubiquitination of several RAS-related guanosine triphosphatases (GTPases) in human meningeal cells (*21*). Thus, the protein levels of four RAS family proteins including KRAS, NRAS, HRAS, and RRAS were examined in mouse tissues. As shown in Fig. 3A, the protein levels of KRAS and NRAS were markedly elevated in brain and heart tissues from *Traf7*^R654Q/+^ mice compared with those from WT littermates. In contrast, the protein levels of HRAS and RRAS remained unchanged (Fig. 3, A to C). Consistently, the protein levels of KRAS and NRAS, but not HRAS and RRAS, were markedly up-regulated in *Traf7*^R654Q/+^ PCNs compared with those in WT PCNs (Fig. 3D). Notably, the mRNA levels of *Kras* and *Nras* were comparable in brain and heart tissues between *Traf7*^R654Q/+^ mice and WT littermates (Fig. 3, G and H), indicating that TRAF7 may play a role in the posttranscriptional regulation of these proteins. Collectively, these results indicate that the MAPK-ERK1/2 pathway is hyperactivated in *Traf7*^R654Q/+^ mice.

### CAFDADD-associated TRAF7 mutants are defective in promoting ubiquitination and degradation of K/NRAS

**Fig. 4.**
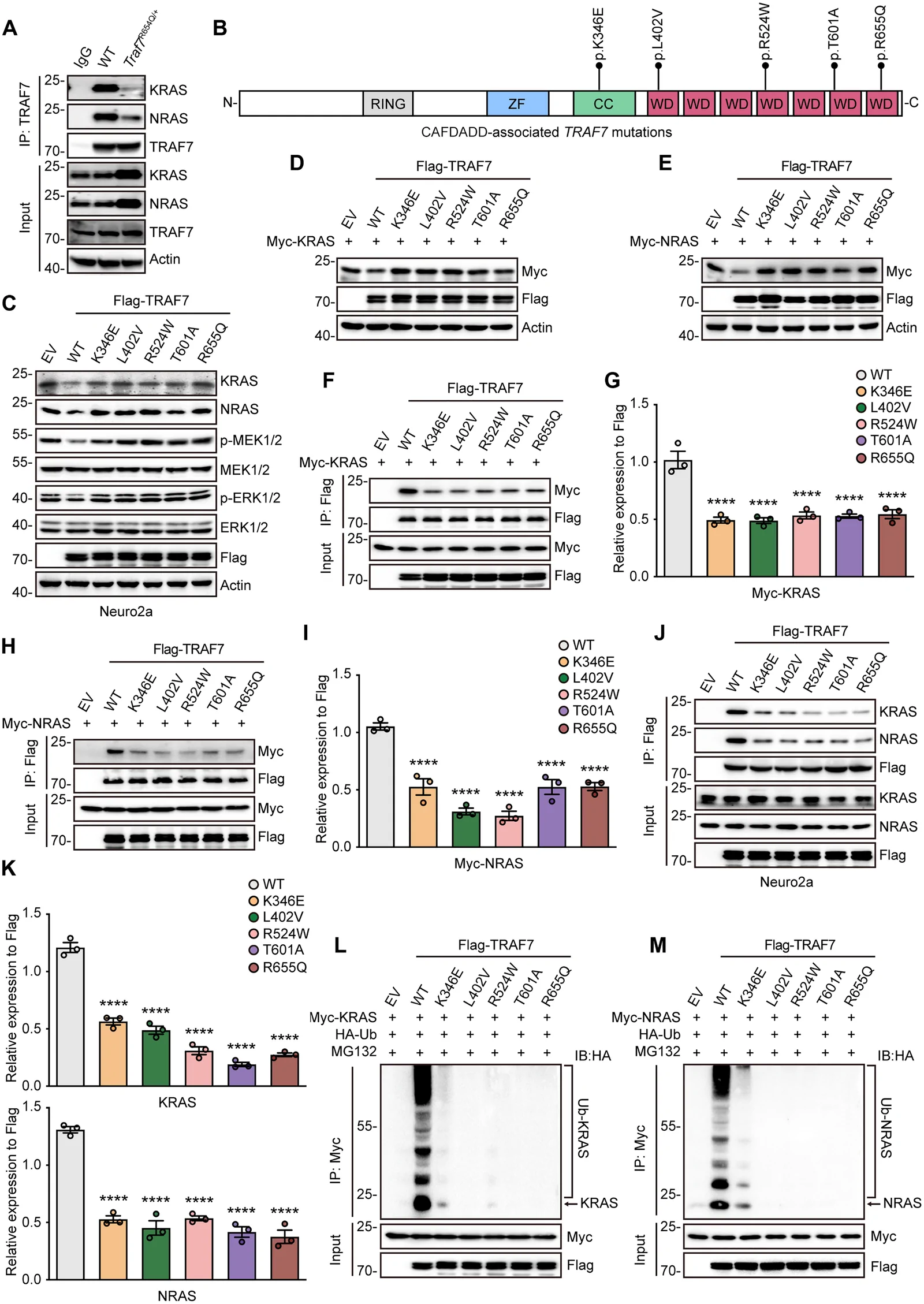
CAFDADD-associated TRAF7 mutants are defective in promoting ubiquitination and degradation of K/NRAS. (**A**) The WCLs of brain tissues in *Traf7*^R654Q/+^mice were prepared and subjected to endogenous co-IP with anti-TRAF7 antibody, and the immunoprecipitates were further analyzed by WB with indicated antibodies. (**B**) Schematic representation of five CAFDADD-associated *TRAF7* mutations occurring in the CC and WDR domains. ZF, zinc finger. (**C**) WB analysis of WCLs from Neuro2a cells transfected with indicated plasmids. (**D** and **E**) WB analysis of WCLs from human embryonic kidney (HEK) 293T cells transfected with indicated plasmids. (**F** to **I**) The WCLs from HEK293T cells transfected with indicated plasmids were prepared and subjected to co-IP with anti-Flag antibody, followed by WB analysis with indicated antibodies. Quantification of coprecipitated KRAS (G) and NRAS (I) from (F) and (H), respectively, is normalized to the intensity of Flag. (**J** and **K**) The WCLs from Neuro2a cells transfected with indicated plasmids were prepared and subjected to co-IP with anti-Flag antibody, followed by WB analysis with indicated antibodies. The intensity of coprecipitated proteins is normalized to the intensity of Flag. (**L** and **M**) In vivo ubiquitination assays in HEK293T cells transfected with indicated plasmids, analyzed by WB. Data are presented as means *±* SEM. One-way ANOVA tests were used in (G), (I), and (K). ****P < 0.0001.

So far, a total of 31 heterozygous mutations in the *TRAF7* gene have been identified in 96 affected CAFDADD individuals (fig. S6 and table S1). All these variants are missense mutations. The majority of them (24 of 31; 77.4%) are located within the WDR domain, followed by the CC domain (7 of 31; 22.6%) (fig. S6). Given that the WDR domain is one of the most prevalent protein interaction modules (*22*), we hypothesized that TRAF7 interacted with K/NRAS through its WDR domain, and those pathogenic mutations disrupt this specific function. To validate this hypothesis, we first constructed a TRAF7 plasmid lacking the WDR domain and performed an exogenous coimmunoprecipitation (co-IP) assay along with TRAF7-WT. TRAF7 was found to strongly interact with K/NRAS, whereas deletion of the WDR domain virtually abolished the interaction between TRAF7 and K/NRAS (fig. S5, B and C). Furthermore, endogenous co-IP assays performed on mouse brain tissues revealed that the Traf7^R654Q^ mutant protein markedly reduced the interaction with K/NRAS compared with Traf7-WT (Fig. 4A), indicating the decreased binding affinity between the mutant TRAF7 and these proteins. The R655Q mutant and TRAF7-WT were found to form heterodimers (fig. S5D), indicating a potential mechanism for the effects of germline heterozygous variants. Dose-dependent co-IP assays showed that WT-R655Q heterodimers interacted more weakly with K/NRAS, suggesting a dominant-negative effect of CAFDADD-associated mutants that impaired the binding ability of the WT protein even at low levels of expression (fig. S5, E and F).

To further define the mechanism, we generated a panel of CAFDADD-associated TRAF7 mutants, including K346E, L402V, R524W, T601A, and R655Q (equivalent to R654Q in mouse) (Fig. 4B). These mutants, along with TRAF7-WT, were overexpressed in Neuro2a cells. We observed that overexpression of TRAF7-WT resulted in a remarkable down-regulation of endogenous K/NRAS protein levels and reduced phosphorylation levels of MEK1/2 and ERK1/2, whereas none of the CAFDADD-associated TRAF7 mutants exhibited similar effects (Fig. 4C). In addition, these mutants were also defective in promoting the down-regulation of ectopically overexpressed KRAS or NRAS (Fig. 4, D and E). Exogenous or semi-endogenous co-IP assay results indicated that the CAFDADD-associated TRAF7 mutants exhibited a reduced binding affinity to K/NRAS compared with TRAF7-WT (Fig. 4, F to K). These findings were consistent with the observed reduction in TRAF7-K/NRAS interaction in *Traf7*^R654Q/+^ mouse brain tissues. Last, in vivo ubiquitination assay results demonstrated that TRAF7-mediated ubiquitination of K/NRAS was almost completely abolished in the presence of these mutants (Fig. 4, L and M).

Overall, these findings indicate that the WDR domain–mediated interaction between TRAF7 and K/NRAS is essential for TRAF7-based ubiquitination and subsequent degradation of K/NRAS. The CAFDADD-associated TRAF7 mutants that we examined generally act in a dominant-negative manner, leading to dysfunction of TRAF7 protein and K/NRAS stabilization.

### *TRAF7*^R655Q/+^ hCOs derived from patients with CAFDADD exhibit early cortical defects

The sMRI analysis revealed significant volume alterations across multiple brain regions, with distinctive volume reductions specifically in the cortical regions of *Traf7*^R654Q/+^ mice (Fig. 2L). Given these observations, we focused our analysis on cortical development using an hCOs model generated from our patient-derived hiPSC line (*TRAF7*^R655Q/+^) and a control line from her healthy father (*18*) and further successfully generated hCOs (fig. S7, A and B). To further ensure that the identified developmental disruptions are specifically driven by the *TRAF7* variant and minimize confounding effects of genetic background, we generated an isogenic “correction” (sg-correction) iPSC line using CRISPR-Cas9 gene-editing technology, as an additional control. Successful correction of the R655Q mutation back to the WT sequence was confirmed by polymerase chain reaction (PCR) followed by Sanger sequencing (fig. S7, C and D). Subsequently, we modeled the disease in a set of cortical neural induction experiments.

**Fig. 5.**
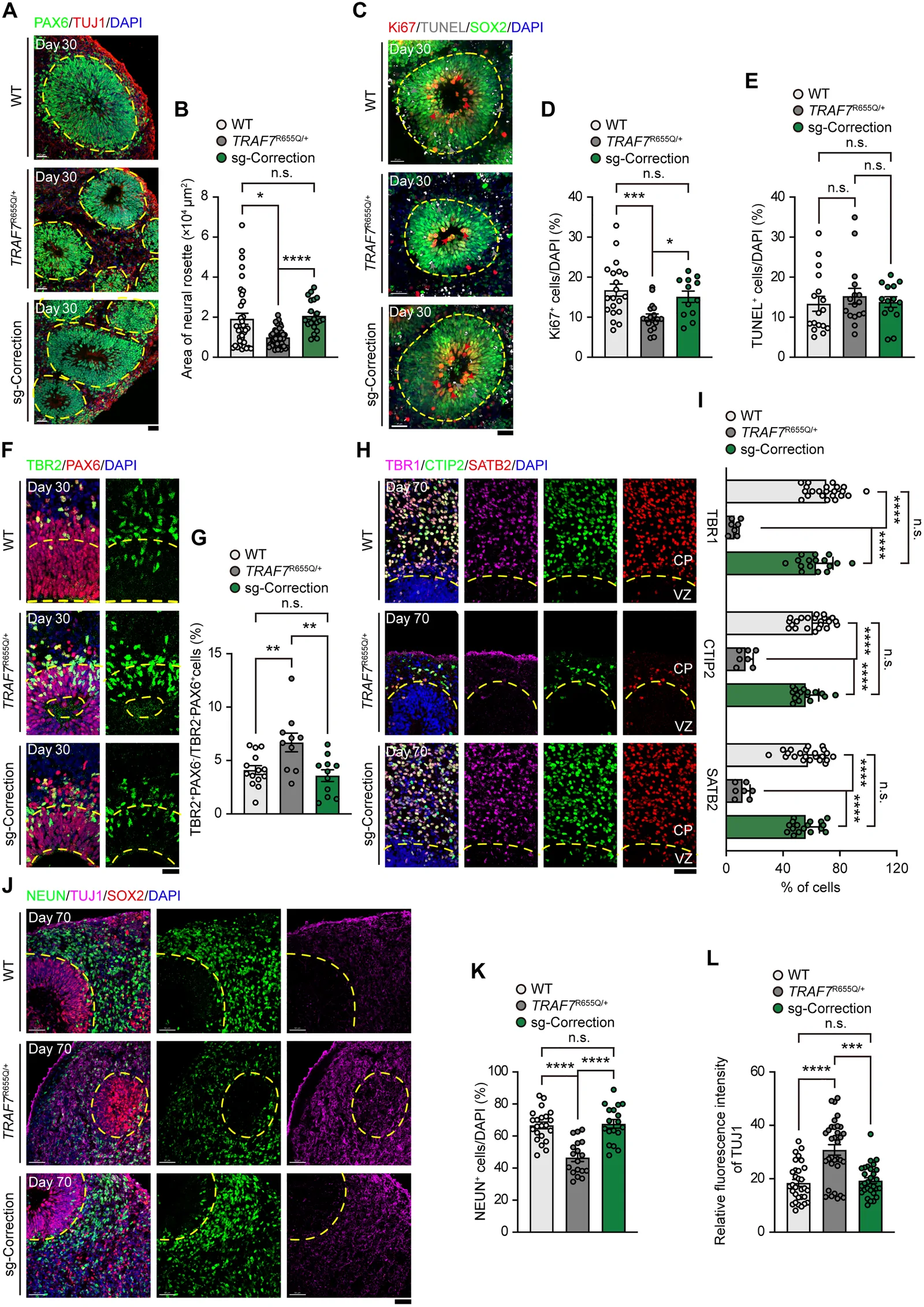
*TRAF7*^R655Q/+^ hCOs derived from patients with CAFDADD exhibit early cortical defects. (**A** and **B**) Immunostaining with PAX6 and TUJ1 antibodies outlines the neural tubes (yellow dashed lines) of hCOs on day 30. The area of neural rosettes is compared in (B) (*n* = 6). The circles in the statistical graph represent the number of neural rosettes that were counted. Scale bar, 30 μm. (**C** to **E**) Reduced proliferation and unchanged apoptosis in *TRAF7*^R655Q/+^ hCOs (*n* = 6) on day 30. The circles in the statistical graph represent the number of VZ regions that were counted. VZ regions are outlined by yellow circle. Ki67^+^ cells (red arrows) and TUNEL^+^ cells (gray arrows) are indicated. Scale bar, 30 μm. (**F** and **G**) Increased TBR2^+^PAX6^−^ cells in *TRAF7*^R655Q/+^ hCOs (*n* = 6) on day 30. The circles in the statistical graph represent the number of fields that were counted. Scale bar, 30 μm. (**H** and **I**) Reduced cortical layer neurons in *TRAF7*^R655Q/+^ hCOs (*n* = 6) on day 70. Immunostaining for TBR1, CTIP2, and SATB2 (H) and their quantification (I). The circles in the statistical graph represent the number of CP regions that were counted. Scale bar, 50 μm. (**J** to **L**) Altered neuronal maturation in *TRAF7*^R655Q/+^ hCOs (*n* = 6) on day 70. The percentage of NEUN^+^ cells (K) and the intensity of TUJ1^+^ staining (L) are shown. The circles in the statistical graph represent the number of CP regions that were counted. Scale bar, 50 μm. Data are presented as means *±* SEM. One-way ANOVA tests or Kruskal-Wallis *H* tests were used as appropriate in (B), (D), (E), (G), (K), and (L). Two-way ANOVA tests were used in (I). *P < 0.05, **P < 0.01, ***P < 0.001, ****P < 0.0001; n.s., not significant. DAPI, 4′,6-diamidino-2-phenylindole.

We monitored the growth of hCOs throughout the culture period and characterized their expansion dynamics over 35 days based on the projected two-dimensional (2D) surface area of organoids (fig. S7E). After 19 days of culture, *TRAF7*^R655Q/+^ hCOs exhibited a statistically significant reduction in overall growth compared with both control groups, including WT and sg-correction counterparts (fig. S7E). Immunostaining with neural progenitor cells (NPCs) markers SOX2 and PAX6 revealed that the neural tube structures assembled with neuroepithelial cells. On day 30, PAX6^+^ NPCs and TUJ1^+^ neurons delineated the neural rosettes, corresponding to the central ventricular zone (VZ)–like zones in the *TRAF7*^R655Q/+^ and control groups. Compared with the control groups, *TRAF7*^R655Q/+^ hCOs exhibited smaller neural tubes, suggesting a reduction of neuroepithelial cells with the *TRAF7*^R655Q^ mutation (Fig. 5, A and B). Assessment of neuroepithelial cell proliferation and apoptosis using Ki67 staining and terminal deoxynucleotidyl transferase–mediated deoxyuridine triphosphate nick end labeling (TUNEL) staining revealed a significantly reduced number of Ki67^+^ cells in *TRAF7*^R655Q/+^ hCOs compared with that of the control groups, whereas the proportion of apoptotic cells remained unchanged on day 30 (Fig. 5, C to E). During cortical development, cortical neurons in the fetal cortex are generated through a two-step process: NPCs localized in the VZ divide to generate TBR2-expressing intermediate progenitor cells that migrate to the subventricular zones where they divide and differentiate into cortical neurons (*23*, *24*). Using TBR2 as a marker for neural differentiation, we found a significant increase in TBR2^+^PAX6^−^ cells during the early development of *TRAF7*^R655Q/+^ hCOs compared with that in the control groups (Fig. 5, F and G). This finding suggests that NPCs tend to undergo the neural differentiation earlier during cortical development in the presence of the *TRAF7*^R655Q^ mutation.

To determine whether premature neurogenesis and the deficiency of proliferative progenitor pools affected cortical neuron differentiation in *TRAF7*^R655Q/+^ hCOs, we explored the cortical plate (CP) on day 70 using cortical layer–specific markers including TBR1 (layer VI), CTIP2 (layer V), and SATB2 (layers II to IV). These markers largely covered the areas surrounding to the VZ region, delineating the CP-like regions within the control hCOs (Fig. 5H). However, quantitative analysis of the immunostaining results revealed a significant reduction in the proportions of all three cortical projection neuron subtypes within the CP region of *TRAF7*^R655Q/+^ hCOs (Fig. 5, H and I). Further analysis using TUJ1 (a pan-neural marker) and NEUN (a mature neuron marker) on day 70 showed decreased NEUN^+^ cells in *TRAF7*^R655Q/+^ hCOs, despite an increase in TUJ1 expression (Fig. 5, J to L), suggesting incomplete or delayed cortical neuron maturation. Furthermore, multielectrode array recordings on hCOs revealed impaired electrophysiological activity in the neuronal networks of *TRAF7*^R655Q/+^ hCOs (fig. S8, A and B). Specifically, compared with the control groups, the mutant hCOs exhibited a significantly reduced mean firing rate and mean spike amplitude, along with a marked decrease in the number of spikes per burst and a loss of synchronized network bursts (fig. S8, C to F), implying the potential functional abnormalities triggered by delayed neuronal maturation in mutant hCOs. Collectively, these results suggest that *TRAF7* mutations disrupt the growth trajectory of NPCs and function of cortical neurons, potentially contributing to early cortical defects.

### MAPK-ERK1/2 signaling is constitutively activated in *TRAF7*^R655Q/+^ hCOs derived from patients with CAFDADD

**Fig. 6.**
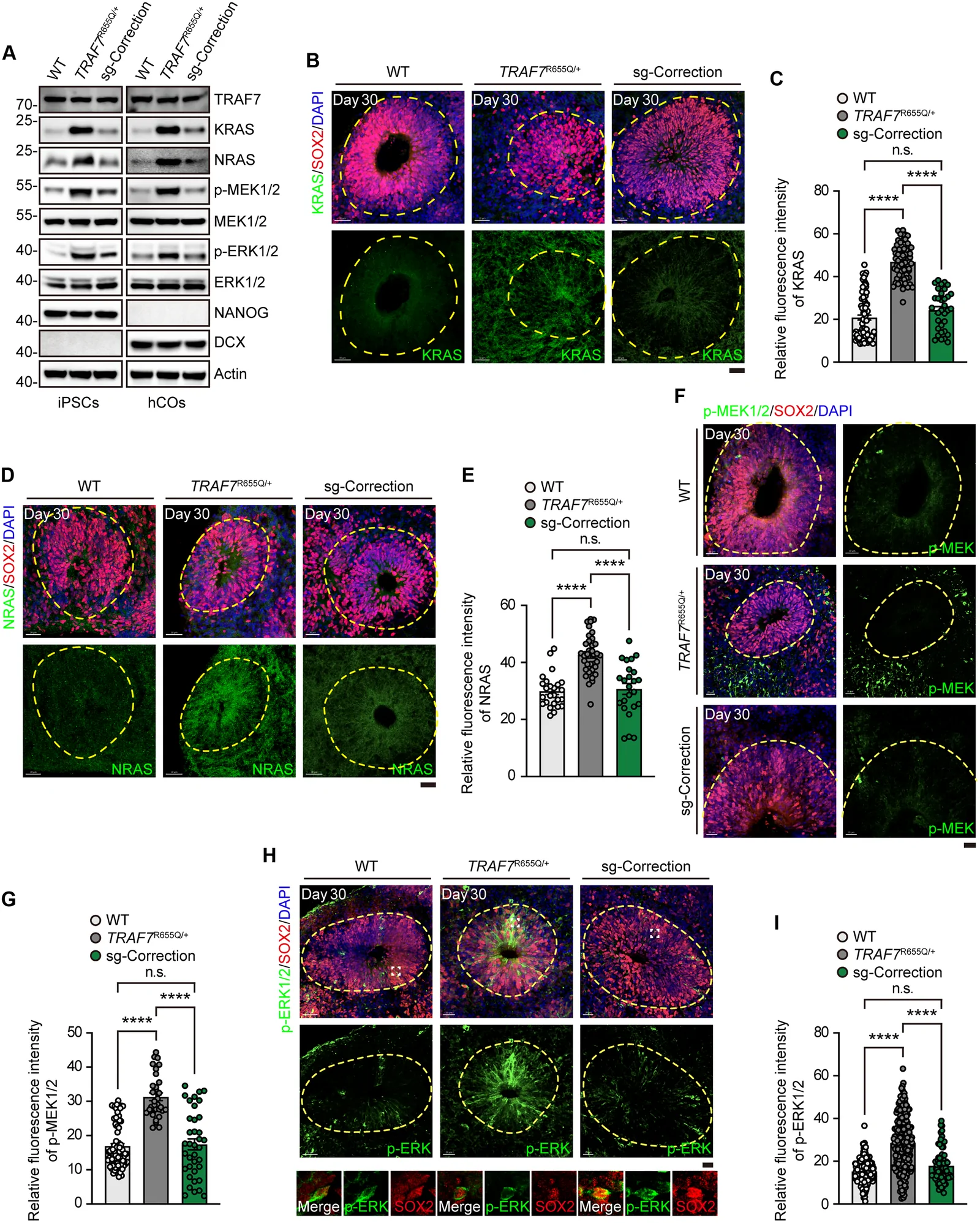
MAPK-ERK1/2 signaling is constitutively activated in *TRAF7*^R655Q/+^ hCOs derived from patients with CAFDADD. (**A**) WB analysis of WCLs from iPSCs and hCOs with indicated antibodies. (**B** and **C**) Comparison of the intensity of KRAS staining in the hCOs (*n* = 5). The circles in the statistical graph represent the number of hCO slices that were counted. Scale bar, 30 μm. (**D** and **E**) Comparison of the intensity of NRAS staining in the hCOs (*n* = 5). The circles in the statistical graph represent the number of hCO slices that were counted. Scale bar, 30 μm. (**F** and **G**) Comparison of the phosphorylation intensity of MEK1/2 staining in the hCOs (*n* = 5). The circles in the statistical graph represent the number of hCO slices that were counted. Scale bar, 20 μm. (**H** and **I**) Comparison of the phosphorylation intensity of ERK1/2 staining in the hCOs (*n* = 5). The circles in the statistical graph represent the number of hCO slices that were counted. Scale bar, 20 μm. Data are presented as means *±* SEM. One-way ANOVA tests or Kruskal-Wallis *H* tests were used as appropriate in (C), (E), (G), and (I). ****P < 0.0001; n.s., not significant.

The MAPK-ERK1/2 pathway plays a critical role in neurodevelopment (*16*), with ERK1/2 pathway activation promoting the differentiation of NPCs into neurons (*25*, *26*) through multiple mechanisms, such as increasing Wnt expression (*27*) or inhibiting BMP/SMAD1 signaling (*28*). Using *TRAF7*-mutant models, we found that *TRAF7* mutations increase K/NRAS stability, leading to activation of the MAPK signaling pathway. We next investigated whether MAPK-ERK1/2 signaling is similarly hyperactivated in patient-specific iPSCs or hCOs. Both *TRAF7*^R655Q/+^ iPSCs and hCOs exhibited higher phosphorylation levels of MEK1/2 and ERK1/2 compared with their respective controls, including WT and sg-correction counterparts (Fig. 6A). Parallel increases in K/NRAS were also observed in both *TRAF7*^R655Q/+^ iPSCs and hCOs (Fig. 6A), which were consistent with the increased MAPK-ERK1/2 cascade previously observed in *Traf7*^R654Q/+^ mice. To further characterize the RAS-MAPK pathway, we performed immunostaining analysis of SOX2-labeled NPCs on day 30. The immunostaining results showed that the protein intensities of K/NRAS (Fig. 6, B to E) and the phosphorylation levels of MEK1/2 and ERK1/2 were significantly higher in *TRAF7*^R655Q/+^ hCOs compared with those in the control groups (Fig. 6, F to I). Collectively, these findings further demonstrate that CAFDADD-associated *TRAF7* mutations drive hyperactivation of the MAPK-ERK1/2 signaling pathway in hCOs, identifying this pathway as a key contributor to the pathogenesis of neurodevelopmental abnormalities in CAFDADD.

### Selumetinib substantially mitigates neurodevelopment-related abnormalities in both *TRAF7*^R655Q/+^ hCOs and *Traf7*^R654Q/+^ mice

**Fig. 7.**
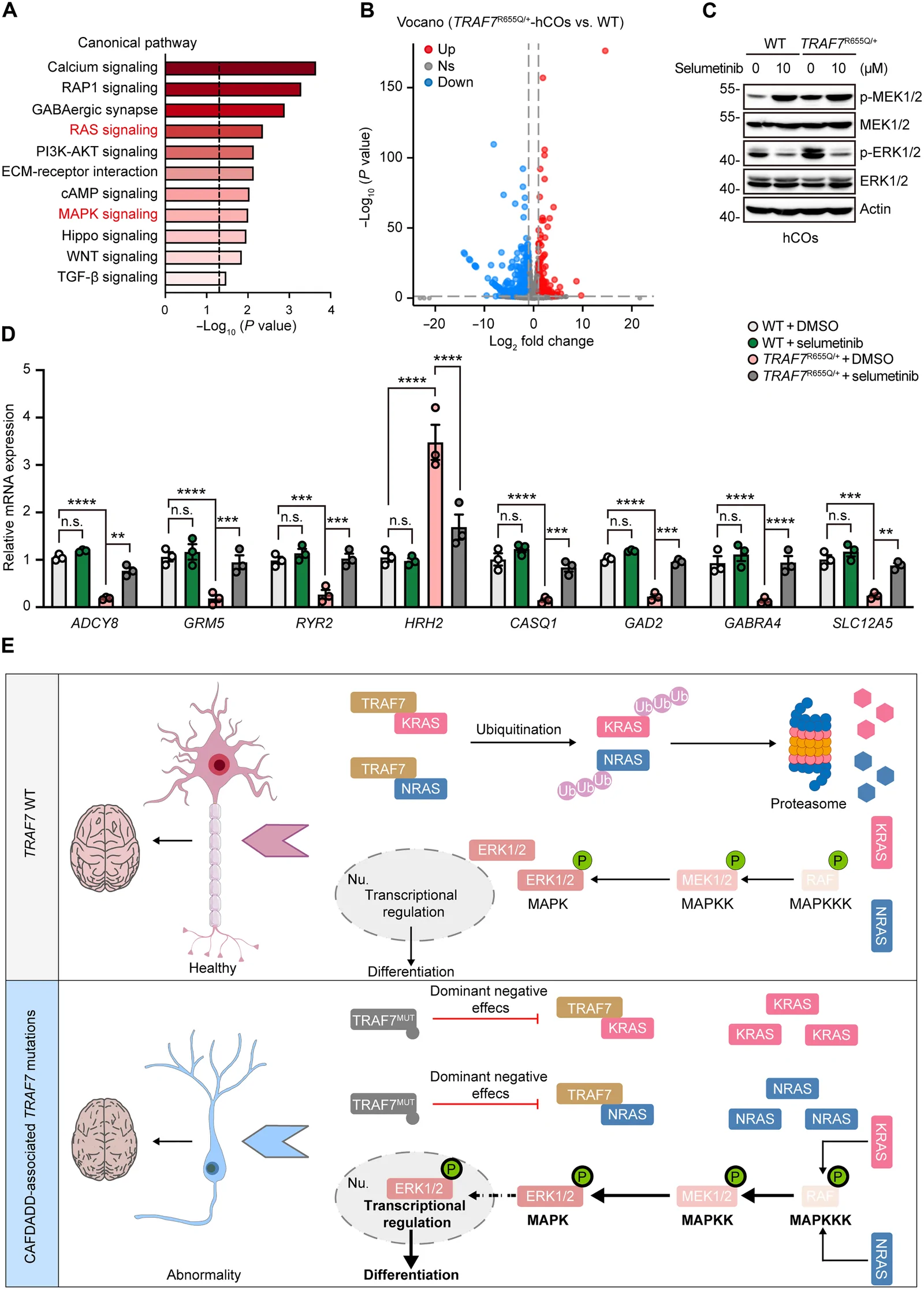
Selumetinib substantially alleviates the transcriptional abnormalities associated with neurodevelopment in *TRAF7*^R655Q/+^ hCOs. (**A**) Ingenuity pathway analysis of DEGs in *TRAF7*^R655Q/+^ hCOs, showing selected overrepresented canonical pathways. The number of DEGs in each pathway is specified. A −log_10_ (*P* value) > 1.3 was considered significant. PI3K, phosphatidylinositol 3-kinase; ECM, extracellular matrix; cAMP, cyclic adenosine 3′,5′-monophosphate; TGF-β, transforming growth factor–β. (**B**) Volcano plots represent DEGs in hCOs, measuring changes in expression (log_2_ fold change) and their significance [−log_10_ (*P* value) > 1.3]. Up: log_2_ fold change > 1 and −log_10_ (*P* value) > 1.3. Down: log_2_ fold change < −1 and −log_10_ (*P* value) > 1.3. Ns: |log_2_ fold change| < 1 or −log_10_ (*P* value) < 1.3. (**C**) WB analysis of indicated proteins in WCLs from hCOs treated with 10 μM selumetinib for 48 hours. DMSO, dimethyl sulfoxide. (**D**) Selumetinib partially restored indicated genes involved in the calcium and GABAergic synapse pathways in *TRAF7*^R655Q/+^ hCOs, measured by RT-qPCR. (**E**) A schematic diagram is illustrated to depict how CAFDADD-associated *TRAF7* mutations lead to K/NRAS stabilization and MAPK-ERK1/2 hyperactivation. Data are presented as means *±* SEM. Two-way ANOVA tests were used in (D). **P < 0.01, ***P < 0.001, ****P < 0.0001; n.s., not significant.

To further investigate the impact of *TRAF7* mutations on gene expression, we performed whole-transcriptomic mRNA sequencing analysis of hCOs. Based on the thresholds of *P* < 0.05 and |log_2_ fold change| ≥ 1, we identified 560 DEGs under basal conditions, comprising 185 up-regulated genes and 375 down-regulated genes (fig. S9, A and B, and table S2). KEGG pathway analysis of the *TRAF7*^R655Q^ transcriptome revealed significant enrichment in the RAS and MAPK canonical pathways. Target DEGs included Ras GTPase-activating protein *RASA4*, several kinases (*PDGFRA*, *MET*, *TEK*, *PRKCB*, and *ERBB3*), protein signaling molecules (*IGF2*, *BDNF*, *FGF4*, *EGF*, *PGF*, *KSR2*, and *CACNG3*), and transcription factors (*MEF2C*) (Fig. 7, A and B; fig. S9C; and table S3), confirming that *TRAF7* mutations cause transcriptional dysregulation of RAS-MAPK signaling. Additionally, multiple neurodevelopment-associated DEGs related to calcium and GABAergic synapse pathways were also enriched in the KEGG analysis (Fig. 7, A and B; fig. S9D; and table S3), suggesting altered neuronal network function in *TRAF7*^R655Q/+^ hCOs. Other significantly enriched canonical pathways, including the phosphatidylinositol 3-kinase–AKT, Hippo, Wnt, and transforming growth factor–β signaling pathways, all of which are crucial for NPC differentiation and maturation (Fig. 7A). Gene ontology analysis further linked these pathways to development and function of the skeletal, nervous, and cardiovascular systems, as well as in behaviors, neuronal projection, and calcium-release channel activity (fig. S9E and table S4). These findings suggest that germline *TRAF7* mutations disrupt the key transcriptional regulatory pathways in neurodevelopment, potentially driving neurodevelopmental abnormalities observed in CAFDADD.

We next explored whether pharmacological inhibition of the hyperactivated K/NRAS-MAPK-ERK1/2 pathway could ameliorate the transcriptional dysregulation caused by the *TRAF7*^R655Q^ mutation in hCOs. Two different neuronal cell lines were treated with increasing concentrations of selumetinib, a clinically approved MEK1/2 inhibitor for NF1 therapy (*29*). Selumetinib treatment markedly reduced the phosphorylation levels of ERK1/2 in a dose-dependent manner (fig. S10, A and B). Based on these results, 10 μM selumetinib was selected to treat hCOs on day 45, which similarly decreased phosphorylation levels of ERK1/2 as confirmed by WB (Fig. 7C). The concomitant compensatory increase in p-MEK1/2 levels likely reflects the well-documented feedback reactivation of upstream signaling upon MEK inhibition rather than a loss of drug efficacy (Fig. 7C and fig. S10, A and B) (*30*, *31*). Notably, reverse transcription quantitative PCR (RT-qPCR) analysis validated that selumetinib treatment significantly normalized the expression of eight DEGs (*BDNF*, *PDGFRA*, *TEK*, *MET*, *EGF*, *PRKCB*, *FGF4*, and *PGF*) associated with the hyperactivity of the RAS-MAPK pathways in *TRAF7*^R655Q/+^ hCOs (fig. S10C). *BDNF*, *PDGFRA*, and *EGF* are key factors in the neurodevelopmental process (*32*–*34*). Selumetinib treatment partially corrected the expression levels of key neurogenesis-related DEGs involved in calcium and GABAergic synapse pathways, which are both critical for neocortical development, including *ADCY8*, *GRM5*, *RYR2*, *HRH2*, *CASQ1*, *GAD2*, *GABRA4*, and *SLC12A5* (Fig. 7D).

Subsequently, we treated hCOs with increasing concentrations of selumetinib below 10 μM to determine an appropriate dose for long-term exposure while minimizing potential toxic effects (fig. S10D). Based on these results, 5 μM selumetinib was selected to treat *TRAF7*^R655Q/+^ and isogenic sg-correction hCOs from days 19 to 30. Compared with its isogenic control, selumetinib significantly rescued the reduced size of *TRAF7*^R655Q/+^ hCOs on day 30 (fig. S10, E and F). WB analysis further confirmed a robust reduction in ERK1/2 phosphorylation in both groups upon selumetinib treatment (fig. S10G). To investigate the impact on neural differentiation, we performed TBR2 and PAX6 coimmunostaining and found that selumetinib statistically reduced the aberrant accumulation of TBR2^+^PAX6^−^ progenitors in *TRAF7*^R655Q/+^ hCOs compared with those in the isogenic controls (fig. S10, H and I). Concurrently, TUNEL staining demonstrated that selumetinib did not significantly increase neuroepithelial cell apoptosis, indicating no obvious cytotoxicity under these therapeutic conditions (fig. S10, J and K). To extend these observations in vivo, pregnant *Traf7*^R654Q/+^ mice (*n* = 3) were treated with selumetinib via daily intraperitoneal injections (2 mg/kg) from E3.5 to P9, followed by direct intraperitoneal administration to the pups every other day until P21. Although no homozygous offspring were recovered at P9, selumetinib treatment partially mitigated the reduced brain weight-to-body weight ratio in *Traf7*^R654Q/+^ mice (fig. S10, L and M). Moreover, WB analysis of cortical tissues harvested from these mice revealed a marked reduction in ERK1/2 phosphorylation (fig. S10N), confirming the effective in vivo suppression of hyperactivated MAPK signaling. In summary, these preclinical evaluations demonstrate that MEK1/2 inhibition substantially ameliorates *TRAF7*-associated abnormalities across molecular, cellular, and in vivo developmental readouts.

## DISCUSSION

Despite substantial progress in establishing the genetic association between germline *TRAF7* mutations and CAFDADD, the molecular mechanisms underlying disease development remain poorly understood, largely due to the lack of relevant experimental models. In this study, we established multiple CAFDADD-associated models and demonstrated that TRAF7 functions as a critical posttranslational regulator of RAS proteostasis by promoting the ubiquitin-mediated proteasomal degradation of KRAS and NRAS, thereby maintaining dynamic homeostatic control over downstream ERK1/2 signaling. CAFDADD-associated TRAF7 mutants exert a dominant-negative effect that disrupts RAS protein turnover, leading to the posttranslational stabilization of K/NRAS and consequent constitutive activation of the MAPK-ERK1/2 pathway in *Traf7*^R654Q/+^ mice, *TRAF7*^R655Q/+^ hCOs, and multiple *TRAF7*-mutant cellular models. Crucially, our newly established KI mouse model successfully and partially recapitulates the multisystemic developmental defects seen in human patients harboring these recurrent germline mutations, including abnormalities of brain, heart, limbs, and skeletal muscle. Together with complementary hCO models, these experimental systems establish a pivotal mechanistic link between impaired RAS proteostasis and CAFDADD-associated abnormalities while providing a valuable preclinical platform for evaluating pathway-targeted pharmacological strategies (Fig. 7E).

The target disruption of *Traf7* in mice illuminates its complex regulatory dynamics during embryogenesis. While biallelic mutations lead to early lethality akin to complete gene deletion (*7*), heterozygous *Traf7*^R654Q/+^ mice survive postnatally but display a broad spectrum of developmental abnormalities. These defects include craniofacial and skeletal abnormalities, marked growth retardation, myocardial dysplasia, limb defects, age-related ocular manifestations alongside intrinsic learning, memory, locomotor, and social deficits. In contrast, *Traf7*^+/−^ mice are phenotypically normal and fertile, whereas heart defects are only present in *Traf7*^−/−^ mice (*7*). These mouse models collectively establish that a single functional *Traf7* allele is insufficient when a dominant-negative variant is present. It should be noted that our *Traf7*^R654Q/+^ mouse model has distinct phenotypic boundaries and does not capture the full spectrum of disease manifestations observed in CAFDADD, particularly specific cardiac defects such as PDA (*4*). Such discordances may reflect contributions from species-specific effects, differences in developmental context, and potential genetic modifiers that influence phenotypic expressivity in monogenic developmental disorders (*35*), underscoring the need for caution when extrapolating from model systems to human disease. Nevertheless, our mouse model shares the genetic background of patients with CAFDADD and partially reproduces multisystem phenotypes, thereby providing a reliable, tractable in vivo tool to dissect the molecular trajectories underlying TRAF7-mediated developmental syndrome.

Among the multiorgan involvements that potentially contribute to CAFDADD-associated behavioral impairments, abnormal brain development is one of the primary symptoms observed in *Traf7*^R654Q/+^ mice. Brain structural changes in *Traf7*^R654Q/+^ mice, including alterations in the motor cortex, HP, HT, LVE, and CBC, were identified via sMRI, with these brain regions also showing high Traf7 expression. Consistently, *TRAF7*^R655Q/+^ hCOs showed smaller size and neural tubes, which are similar to the reduction in brain size of *Traf7*^R654Q/+^ mice. Cortical defects in both models are akin to the cerebral atrophy observed in patients with CAFDADD. Aberrant growth trajectories and neuronal differentiation of NPCs in *TRAF7*^R655Q/+^ hCOs, including reduced proliferative NPCs, earlier NPC differentiation, fewer cortical projection neuron subtypes, incomplete or delayed maturation of cortical neurons, and impaired functional activity of neuronal networks, are likely the causes of cortical defects. Together, our study uses hCOs to reveal that CAFDADD-associated *TRAF7* mutations impair early neuronal behavior patterns during embryogenesis, ultimately contributing to abnormal brain development and the resulting neurological and behavioral defects. To safeguard against the stochastic variability inherent to organoid differentiation and control for genetic background effects, we generated a CRISPR-corrected isogenic control line. This pairwise approach ensures that the identified developmental disruptions are specifically driven by the *TRAF7* variant. Nevertheless, we recognize the limitation of using a single patient-derived genetic background. Future longitudinal studies integrating additional independent mutant and matched control lines remain essential to fully account for phenotypic expressivity and to strengthen the generalizability of these neural trajectories.

The MAPK-ERK1/2 signaling pathway plays a crucial role in regulating cellular proliferation and differentiation of nervous tissues in neurodevelopment, with ERK1/2 activity needing to be tightly maintained within a narrow dynamic range (*16*). Clinically, dysregulation of this pathway underlies classical RASopathies and has also been implicated in neurodevelopmental disorders, such as ASD (*17*, *36*). Consistently, overall activation or loss of ERK1/2 in mice causes embryonic lethality, reduced cortical thickness, abnormal behavior, impaired learning and long-term memory, and social deficits (*20*, *37*, *38*), several of which overlap with the phenotypes observed in *Traf7*^R654Q/+^ mice. In this study, our initial findings suggest that the stress-responsive MEKK3-p38/JNK pathway is not responsible for the phenotypes observed in *Traf7*^R654Q/+^ mice. Notably, constitutive hyperactivation of MAPK-ERK1/2 signaling was observed in our model mice and across multiple cell types. Although ERK1/2 signaling has not previously been directly linked to TRAF7, it is a well-established downstream effector of the RAS-RAF-MEK1/2 cascade. We therefore examined whether disease-associated *TRAF7* variants alter upstream RAS regulation and found that mutants affecting either the CC or WDR domain impaired the ubiquitin-mediated clearance of KRAS and NRAS, resulting in their increased protein stability. This posttranslational accumulation of K/NRAS sustains downstream ERK1/2 phosphorylation, offering a biochemical explanation for the phenotypic overlap observed across distinct variants. Moreover, *KRAS* and *NRAS* are well-known oncogenes, and germline mutations in these genes typically result in milder biochemical and functional consequences that are compatible with human embryonic development (*39*). During embryogenesis, genetic interactions between *Kras* and *Nras* contribute to the normal survival of mice (*40*). A subset of patients with CAFDADD exhibits phenotypic overlap with NSDs, including CHDs, facial dysmorphism, and scoliosis (*1*). Consistently, *Traf7*^R654Q/+^ mice display some features similar to those observed in NS models, including *Kras*^V14I^-mutant mice (*41*), *Kras*^G12D^ KI mice (*42*), *Nras*^G12D/+^ mice (*43*, *44*), and *Nras*^I24N^-mutant zebrafish (*45*), all of which are driven by MAPK pathway activation. Together, the abnormal hyperactivation of the MAPK-ERK1/2 signaling pathway provides a possible mechanistic link between CAFDADD and RASopathies.

While CAFDADD shares dysregulation of the MAPK pathway with NSDs, our data do not support a direct equivalence between CAFDADD and canonical RASopathies. Instead, multiple discordant clinical features exist between the two diseases. For instance, intellectual disability in CAFDADD tends to be more severe than that in NS (*13*, *46*). Cardiac manifestations also differ: Pulmonary valve stenosis and hypertrophic cardiomyopathy are the most characteristic heart abnormalities in NS (*13*), whereas CAFDADD is more frequently associated with PDA, as well as septal defects, and valvular defects (*1*, *4*). Lymphatic abnormalities tend to be more frequent in NS and related disorders (*13*); however, these phenotypes have not been systematically reported in CAFDADD. NS is associated with an increased risk for hematologic disorders, such as juvenile myelomonocytic leukemia and myeloproliferative diseases, a symptom not observed in CAFDADD, suggesting distinct disease trajectories. In addition, RASopathies have a higher susceptibility to malignancies (*13*, *47*), while only one older patient with CAFDADD developed meningioma, not seen in younger individuals (*1*). These profound phenotypic differences are likely attributable to the distinct molecular mechanism identified in our study, in which impaired ubiquitination leads to altered RAS protein stability rather than primary mutations within the RAS signaling cascade itself. Although our *TRAF7*-mutant systems capture several neurodevelopmental and molecular features, they do not fully recapitulate the clinical spectrum of patients, especially the intricate cardiac phenotypes. Ultimately, our results suggest that *TRAF7*-associated disease represents a mechanistically related but clinically distinct entity from classical RASopathies, characterized by dysregulated RAS proteostasis and downstream signaling.

It is crucial to recognize that TRAF7 functions as a multitarget E3 ubiquitin ligase regulating a broad array of cellular signaling networks. Somatic *TRAF7* mutations contribute to meningioma development by disrupting MEKK3-JNK signaling (*9*) and impairing the ubiquitination and degradation of CDC42, RAC1, and pan-RAS (*21*). Although most germline *TRAF7* mutations are mutually exclusive to somatic variants, we observed that CDC42 and RAC1 were also stabilized in our *TRAF7*-mutant models, resembling the physiological changes of K/NRAS (fig. S11, A to N). These findings suggest a potential mechanism by which germline *TRAF7* mutations could contribute to an age-dependent risk of meningioma in patients with CAFDADD. Further longitudinal studies and careful monitoring of aging syndromic patients will be needed to determine whether their risk of tumor development is increased and clinically meaningful. TRAF7 promotes the turnover of NEMO and p65 via the ubiquitin-lysosome system (*48*) and facilitates the degradation of p53 or HOXA5 via the ubiquitin-proteasome system (*49*, *50*). These prior findings highlight the broad, multipathway functions of TRAF7 beyond the RAS-MAPK cascade. Although our data establish a mechanistic link between CAFDADD-associated *TRAF7* mutations and RAS-MAPK hyperactivation, we do not exclude the possibility that additional substrates or pathways may also contribute to the diverse developmental and multisystem phenotypes of CAFDADD. Therefore, unraveling the distinct and overlapping roles of these non-RAS substrates during embryogenesis represents an essential step toward fully understanding CAFDADD pathogenesis.

Now, there is no curative treatment for CAFDADD syndrome, underscoring the necessity of deciphering its precise pathogenic pathways. In this study, our preclinical evaluation of selumetinib demonstrated that MEK1/2 inhibition can successfully mitigate specific molecular, transcriptional, and early cellular abnormalities triggered by the *TRAF7* variant, thereby firmly cementing the mechanistic correlation between defective RAS ubiquitination and hyperactivated downstream signaling. Nevertheless, CAFDADD is a congenital developmental disorder with origins in embryogenesis, and established structural defects are unlikely to be reversed by postnatal pathway inhibition. Moreover, TRAF7 may regulate additional substrates beyond RAS proteins, and targeting MAPK signaling alone is unlikely to provide a comprehensive therapeutic rescue. These pharmacological experiments should thus be viewed as a preliminary pathway-targeted intervention to validate MAPK-ERK1/2 hyperactivation as one downstream consequence of *TRAF7* mutations, rather than as evidence for a definitive treatment strategy. In summary, although important limitations remain, our study establishes tractable *TRAF7*-mutant in vitro and in vivo models that provide valuable platforms for evaluating pathway-specific or combination strategies, defining safe early-intervention windows, and assessing higher-order neurodevelopmental outcomes.

## MATERIALS AND METHODS

### Generation and breeding of *Traf7*^R654Q^-KI mice

The murine *Traf7* gene (NCBI reference sequence: NM_001172113.2) is located on mouse chromosome 17. *Traf7*^R654Q^ KI mice were generated carrying the p.R654Q mutation (c.1961-1962GG>AA). The model was generated by Cyagen Biosciences (Suzhou, China) using the ES cell targeting technique. The modified PUC19 vector was designed to insert a self-deletion anchor flanked neomycin resistance (Neo) cassette in reverse orientation into the intron 14 of murine *Traf7* via homologous recombination. The p.R654Q point mutation was introduced into exon 20 in the 3′ homology arm. This targeting vector was linearized and transfected into C57BL/6N ES cells according to Cyagen’s standard electroporation procedures. The transfected ES cells were subjected to G418 selection (200 μg/ml) 24 hours postelectroporation. The potential positive clones from PCR screening were expanded and further characterized by Southern blot analysis. One correctly targeted clone was injected into C57BL/6N blastocysts, and male chimeras were obtained. Germline transmission of the targeted mutant allele was verified by crossing the male chimeras to female mice to generate mice with one p.R654Q point mutation allele (F1-*Traf7*^R654Q/+^ mice). Genotyping of F1 pups was performed by PCR amplification of tail DNA followed by Sanger sequencing. Successfully genotyped F1 mice carrying the *Traf7*^R654Q^ mutation were crossed with WT mice to generate F2 offspring. F2 heterozygous mice were identified by genotyping. The sequences used for ES cell targeting technology and the primers used for genotyping (F2 and R2) and sequencing (F1 and R1) of indicated site are provided in table S5.

Mice are maintained under a 12-hour/12-hour light/dark cycle at 22° to 25°C with 40 to 50% humidity and have free access to standard food and water. The offspring of mating pairs are genotyped at P14 and weaned at P21. WT littermates served as controls for *Traf7*^R654Q*/+*^ mice. Invasive operations on animals were performed under anesthesia with pentobarbital sodium (50 mg/kg, intraperitoneal), and all efforts were made to minimize animal suffering. A status of being blind to the genotype was maintained across all procedures during all the assays except biochemical analyses.

All animal experiments were approved by the Ethics Committee of Animal Experimentation at Shanghai Children’s Hospital. All animal care and experimental procedures followed the guidelines of the Care and Use of Laboratory Animals established by the Children’s Hospital of Shanghai and the Shanghai Municipality, P.R. China. The protocol was authorized by the Science and Technology Commission of Shanghai Municipality (permit number SHCH-IACUC-2023-XMSB-65).

### Three-chamber social interaction and social novelty preference test and direct social interaction test

The three-chamber social interaction test was performed according to a previously described protocol (*51*). The apparatus consists of a rectangular box divided into three chambers, with an inverted wire cup placed in each side chamber. In the habituation phase, the test mouse was allowed to freely explore the entire apparatus for 5 min with all chamber doors open. Next, the test mouse was confined to the middle chamber while an unfamiliar mouse of the same sex (stranger 1) was placed under one of the wire cups (the side alternated between tests). The test mouse was then given 5 min to explore the entire apparatus. Next, the test mouse was again confined to the middle chamber, whereas a second unfamiliar mouse of the same sex (stranger 2) was placed under the other wire cup. The test mouse was allowed to explore freely for another 5 min to assess social discrimination. Throughout all phases, the time spent in each chamber and the duration of close interactions with the wire cups were automatically recorded. Social behavior was evaluated by analyzing the time spent in each chamber and the duration of sniffing.

The direct social interaction test was performed following a previously described protocol (*52*). Mice were first habituated in a clean standard cage for 30 min. After habituation, an unfamiliar mouse (stranger A) of the same sex was introduced. Social behaviors initiated by the test subject mouse, including anogenital and nose-to-nose sniffing, following, and allogrooming, were recorded over a 5-min session. Following a 1-hour intertrial interval, the test mice were exposed to either the previously encountered mouse (stranger A) or an unfamiliar mouse (stranger B) for another 5-min session. All mice in the behavioral experiments were randomly assigned to experimental groups. Data analysis was performed in a genotype-blinded manner.

### Barnes maze assay

The Barnes maze consists of a blue circular platform (100 cm in diameter, 80 cm in height) with 20 evenly spaced holes (4 cm in diameter, positioned 2 cm from the edge). The maze is illuminated at 250 lux, and large geometric shapes on the surrounding walls serve as visual cues. A single escape box is positioned beneath a designated target hole. On day 1, mice were given 3 min per trial to explore the maze and locate the escape box. Training was conducted over four consecutive days, with four trials per day. If a test mouse failed to locate the escape box within the allotted time, then it was gently guided to the escape hole. On day 5, the hiding box was removed, and a 100-s probe trial was conducted to assess spatial memory. Spatial memory was evaluated by measuring the latency to reach the escape box during training (days 1 to 4) and the target hole during the probe trial (day 5).

### OF test and OF center test

The apparatus, measuring 40 cm by 40 cm by 40 cm, is illuminated by overhead lighting at 150 lux. Each subject was placed at the center of the apparatus and observed for a 30-min test session. Video recordings were analyzed using EthoVision XT v11.5 (Noldus, USA) to quantify total distance traveled, movement time, and distance traveled within the center area (defined as one-fourth of the total arena). The center distance ratio was calculated as the distance traveled in the center divided by the total distance traveled, representing the proportion of time spent in the center.

### Elevated plus maze

The EPM comprises two open arms and two closed arms extending symmetrically from a central platform. The apparatus has a white floor and transparent walls, is elevated 50 cm above the ground, and is illuminated at ∼100 lux. Each mouse was placed in the central platform facing an open arm and allowed to explore freely for 10 min. Behavior was recorded using a ceiling-mounted camera and analyzed with EthoVision XT v11.5 (Noldus, USA). Anxiety-like behavior was evaluated by the time spent in open and closed arms.

### Echocardiography

WT and *Traf7*^R654Q/+^ mice (8 weeks) were anesthetized using isoflurane inhalation. Cardiac ultrasound imaging was performed using Visual Sonics Vevo 3100 Imaging System. Heart rate and left ventricular dimensions were measured from 2D short-axis under M-mode tracings at the level of the papillary muscle. Cardiac functional parameters, including EF, FS, cardiac output, LVPWd/s, left ventricular anterior wall thickness at end-diastole/systole, left ventricular internal dimension at end-diastole/systole, and heart rate, were automatically calculated using the Vevo 3100 software.

### Structural magnetic resonance imaging

WT and *Traf7*^R654Q/+^ mice (8 weeks) were subjected to a brain sMRI study. Mice were anesthetized with 5% isoflurane for induction and maintained at 1.5 to 2% during imaging. Respiration was continuously monitored, and body temperature was maintained at 37°C using a warm-air feedback system (SA Instruments, USA). In vivo sMRI experiments were performed using a 9.4-T Bruker BioSpec horizontal bore animal scanner (Bruker BioSpin, Ettlingen, Germany) equipped with a gradient system of 660 mT/m and a ramp time of 110 μs. A quadrature volume resonator (inner diameter, 86 mm; Bruker BioSpin) was used for RF excitation, whereas a quadrature mouse brain surface coil (Bruker BioSpin) was used for signal reception. MRI data were acquired using ParaVision 360 software.

### RNA isolation and RT-qPCR

Total RNA was extracted from cells or tissues using the TRIzol reagent (Invitrogen) according to the manufacturer’s protocols. RNA concentrations and purity were assessed using a NanoDrop spectrophotometer (Thermo Fisher Scientific) by measuring ultraviolet (UV) absorbance. First-strand cDNA was synthesized using the HiScript III 1st Strand cDNA Synthesis Kit (Vazyme Biotech), followed by qPCR amplification using ChamQ SYBR qPCR Master Mix (Vazyme Biotech). Relative mRNA expression levels were calculated using the 2^−ΔΔCt^ method, normalized to β-actin. The primer sequences and reagents are provided in tables S5 and S6.

### Cell lines, cell culture, and transfection

Human embryonic kidney 293T (HEK293T) and Neuro2a cells were obtained from the American Type Culture Collection and maintained in Dulbecco’s modified Eagle’s medium (DMEM) supplemented with 10% fetal bovine serum (FBS) and 1% penicillin-streptomycin. Cell line authentication was routinely performed using DNA fingerprinting, and mycoplasma contamination was checked by PCR. The constructed plasmids were transiently transfected into indicated cells using Lipofectamine 2000 (Invitrogen) following the manufacturer’s protocol. Cell lysates were collected 48 hours posttransfection and subjected to SDS–polyacrylamide gel electrophoresis (PAGE) followed by immunoblotting. Details of cell culture conditions, materials, and transfection reagents are provided in table S6.

### Immunoblot analysis

Cells, tissues, and organoids were lysed in ice-cold radioimmunoprecipitation assay buffer [20 mM tris-HCl (pH 7.5), 150 mM NaCl, 1.0 mM EDTA, 1% NP-40, 0.1% SDS, and 0.1% sodium deoxycholate] with 1 mM protease inhibitor cocktail. After sonication on ice, lysates were incubated for 30 min on ice to ensure complete protein extraction. Lysates were centrifuged at 12,000*g* for 20 min at 4°C, and the supernatant was collected and mixed with 5× SDS loading buffer [200 mM tris-HCl (pH 6.8), 400 mM β-mercaptoethanol, 8% SDS, 4.0 mM EDTA, 0.4% bromophenol blue, and 40% glycerol]. A total of 10 to 30 μg protein was separated on 10 or 12% SDS-PAGE gels and transferred onto a 0.22-μm nitrocellulose membrane at 350 mA for 1 hour. Membranes were blocked with 5% bovine serum albumin in phosphate-buffered saline (PBS) containing 0.05% Tween 20 for 1 hour at room temperature, followed by incubation with primary antibodies at 4°C overnight. Horseradish peroxidase–conjugated secondary antibodies [goat anti-rabbit immunoglobulin G (IgG) and goat anti-mouse IgG] were used for detection with an enhanced chemiluminescence kit. Details of the primary and secondary antibodies, as well as other reagents used in this study, are provided in tables S6 and S7.

### In vivo ubiquitination assays

HEK293T cells were transfected with HA-ubiquitin together with the indicated plasmids. At 36 hours posttransfection, cells were treated with 10 μM MG132 for 6 hours before harvest. Cells were lysed in 1% SDS buffer [tris (pH 7.5), 1 mM dithiothreitol, and 0.5 mM EDTA] supplemented with protease inhibitors and 10 mM *N*-ethylmaleimide and boiled for 10 min at 100°C. The lysates were then diluted with IP buffer [20 mM tris-Cl (pH 7.5), 100 mM NaCl, 0.2 mM EDTA, 20% glycerol, and 0.2% Triton X-100] and subjected to immunoprecipitation using anti-Myc antibody prebound to protein A/G magnetic beads. After extensive washing, the immunoprecipitates were analyzed by immunoblotting with an anti-HA antibody to detect ubiquitinated Myc-tagged KRAS or NRAS. Details of the primary and secondary antibodies, as well as other reagents used in this study, are provided in tables S6 and S7.

### PCN culture

PCNs were cultured as previously described (*53*). Briefly, cortical neurons were isolated from P0 mouse pups. The dissociated cells (1 × 10^5^ cells per well) were plated on either six-well plates or glass coverslips precoated with 10 μM poly-d-lysine overnight. The cells were maintained in Neurobasal-A medium supplemented with 5% FBS, B27, 2 mM glutamine, and 1% penicillin-streptomycin for indicated number of days at 37°C in a humidified incubator with 5% CO_2_. Detailed information on cell culture conditions, materials, and reagents is listed in table S6.

### Generation and validation of CRISPR-Cas9–corrected isogenic iPSC line

Patient-derived *TRAF7*^R655Q/+^ iPSCs were subjected to CRISPR-Cas9–mediated genome editing to correct the *TRAF7*^R655Q^ variant. Cells were electroporated with 20 μg of Cas9 protein, 150 pmol of single guide RNA (sgRNA), and a donor plasmid carrying the WT *TRAF7* repair sequence flanked by homology arms and a puromycin-resistance cassette. Following electroporation, edited cells were enriched by puromycin selection (0.5 μg/ml). Single-cell–derived colonies were manually isolated and expanded, after which genomic DNA was extracted from candidate clones. The targeted *TRAF7* locus was amplified by PCR and verified by Sanger sequencing. One clone carrying the corrected WT sequence was selected and used as the sg-corrected isogenic control in subsequent cortical neural induction experiments. The PCR primers and sgRNA target sequence are provided in table S5.

### Generation of hCOs derived from hiPSCs

hiPSCs were successfully derived from PBMCs of a patient with CAFDADD carrying the *TRAF7*^R655Q^ mutation and her healthy father (*18*). The generation of hCOs from these iPSCs was performed as previously described (*54*). To initiate the generation of hCOs, iPSCs cultured on Matrigel were dissociated into intact iPSC colonies using Accutase and transferred to ultralow-attachment plastic 96-well plates in HNM medium [DMEM/F12 with 1:100 N2 supplement, 1% (v/v) GlutaMAX, 1% (v/v) MEM-NEAA, and E8 in a 1:1 ratio], further supplemented with two SMAD inhibitors: 10  μM SB-431542 and 0.1 μM LDN-193189, along with 10 μM Rock inhibitor Y27632. For the first 7 days, the HNM medium was changed daily and supplemented with SB-431542 and LDN-193189. On the eighth day in suspension, the neural spheroids were transferred to neural differentiation medium containing Neurobasal medium, 1:100 N2 supplement, 2% (v/v) B-27 supplement, 1% (v/v) GlutaMAX, 1% MEM-NEAA, and 1% penicillin-streptomycin, supplemented with basic fibroblast growth factor (20 ng/ml) and epidermal growth factor (20 ng/ml). From day 25 onward, hCOs were maintained in neural medium with medium changes every 4 to 6 days. The hCOs were cultured until day 30 or 70, as indicated. We separately counted the sample information presented in multiple fields of view for each type of hCOs. Detailed information on cell culture conditions, materials, and reagents is provided in table S6. All experimental procedures were approved by the Science and Technology Commission of Shanghai Municipality (permit number 2020R007-F01).

### Immunocytochemistry

For fixation, hCOs were incubated in 4% paraformaldehyde for 2 hours at 4°C, followed by three washes with PBS. The organoids were then incubated overnight in a 30% sucrose solution. Organoids were embedded in Tissue Frozen Medium (Leica), frozen in cold isopentane, and sectioned with a cryostat (Leica). For immunostaining, the coverslips were washed three times in PBS and incubated in a blocking solution (0.3% Triton-X and 10% donkey serum in PBS) for 1 hour. Samples were then incubated with indicated primary antibodies overnight at 4°C, followed by three washes in PBS at room temperature before incubation with secondary antibodies. Samples were mounted on glass coverslips for microscopy and imaged using a Leica SP8 Laser scanning confocal microscope and Nikon A1 microscope. The antibodies used in this study are provided in table S7, and image processing was performed using ImageJ software.

### Electrophysiologic data acquisition

We obtained cross-sectional slices from 50-day-cultured hCOs. An organoid slice was transferred to the HD-MEA well and secured to the surface of the electrodes using Matrigel. The well had been precoated with 0.07% (v/v) poly(ethylenimine) (Sigma-Aldrich) in borate buffer, followed by laminin (0.04 mg/ml). The recording medium was prepared as follows: BrainPhys neuronal medium (STEMCELL Technologies), 2% (v/v) NeuroCultTM SM1 Neuronal Supplement (STEMCELL Technologies), 1% (v/v) N2 Supplement-A (STEMCELL Technologies), 1× GlutaMAX, and 1× antibiotic-antimycotic, supplemented with 1 mM dibutyryl-cAMP, brain-derived neurotrophic factor (20 ng/ml), glial cell line–derived neurotrophic factor (20 ng/ml), and NT-3. The recording setup was placed inside a 5% CO_2_ cell culture incubator at 37°C. Recordings were performed using the “Activity scan assay” and “Network assay” modules, featured in the MaxLab Live software (MaxWell Biosystems).

### Whole transcriptional mRNA sequencing library preparation and sequencing

Following procedures previously described in (*55*), we extracted total RNA from hCOs using TRIzol reagent (Invitrogen) according to the manufacturer’s protocols. RNA quality was assessed using the Agilent 5300 Bioanalyzer and quantified with a NanoDrop spectrophotometer (Thermo Fisher Scientific) by measuring UV absorbance. Only high-quality RNA samples [optical density at 260/280 nm (OD_260/280_) = 1.8 to 2.2, OD_260/230_ ≥ 2.0, RQN ≥ 6.5, 28*S*:18*S* ≥ 1.0, and >1 μg] were selected for sequencing library construction. RNA purification, reverse transcription, library preparation, and sequencing were performed by Shanghai Majorbio Bio-pharm Biotechnology Co. Ltd. (Shanghai, China) according to the manufacturer’s instructions. The RNA sequencing (RNA-seq) transcriptome library was prepared using Illumina Stranded mRNA Prep, Ligation (San Diego, CA) with 1 μg of total RNA. Briefly, mRNA was isolated by poly(A) selection with oligo(dT) beads and fragmented. Double-stranded cDNA was synthesized using the Superscript Double-stranded cDNA Synthesis Kit with random hexamer primers. The synthesized cDNA was then subjected to end-repair, phosphorylation, and adapter ligation according to the library construction protocol. Libraries were size selected for cDNA target fragments (∼300 base pairs) on a 2% low-range ultra-agarose gel, followed by PCR amplification using Phusion DNA polymerase for 15 cycles. After quantification using a Qubit 4.0 fluorometer, sequencing was performed on the NovaSeq X Plus platform (PE150) using the NovaSeq Reagent Kit. The raw paired-end reads were trimmed and quality checked using fastp with default parameters. Clean reads were aligned to the reference genome in a strand-specific manner using HISAT2 software, and mapped reads were assembled using StringTie in a reference-based approach.

### Statistical analysis

All quantitative data are presented as means ± SEM from experiments conducted with at least three biological replicates. Statistical analyses were performed using GraphPad Prism (v9.5.1). The normality of data distribution was assessed for each group using the Shapiro-Wilk test, and homogeneity of variance was evaluated using the Brown-Forsythe test. Based on the data distribution and the experimental design, statistical tests were applied as appropriate. For comparisons between two groups, an unpaired two-tailed Student’s *t* test (for parametric data) was used when the assumptions of normality and equal variance were met; otherwise, the Mann-Whitney *U* test (for nonparametric data) was applied. Comparisons across multiple groups under a single factor were performed using one-way analysis of variance (ANOVA) followed by Tukey’s post hoc test (parametric) or the Kruskal-Wallis *H* test followed by Dunn’s post hoc test (nonparametric). For datasets involving two independent factors, a two-way ANOVA test was used. A *P* value of less than 0.05 was considered statistically significant. Statistical significance is indicated as follows: * represents *P* < 0.05; ** represents *P* < 0.01; *** represents *P* < 0.001; **** represents *P* < 0.0001; and n.s. represents not significant (*P* ≥ 0.05).
