# Supplementary material for: TRAF7 mutations stabilize K/NRAS and hyperactivate MAPK signaling to cause CAFDADD neurodevelopmental defects
Source: Sci Adv. 2026 Sep 25;12(39):eadw8672. doi: 10.1126/sciadv.adw8672 (PMC13614401; doi:10.1126/sciadv.adw8672)
Supplement: Supplementary file 1 [file sciadv.adw8672_sm.pdf]

Science Advances
AAAS

Supplementary Materials for

TRAF7 mutations stabilize K/NRAS and hyperactivate MAPK signaling to cause CAFDADD neurodevelopmental defects

Man Xiao *et al.*

Corresponding author: Chenji Wang, chenjiwang@fudan.edu.cn; Lixiang Ma, lxma@fudan.edu.cn; Nan-Jie Xu, xunanjie@sjtu.edu.cn; Shengnan Wu, wushengnan@shchildren.com.cn

Sci. Adv. **12**, eadw8672 (2026)
DOI: 10.1126/sciadv.adw8672

The PDF file includes:

Figs. S1 to S11
Legends for tables S1 to S7

Other Supplementary Material for this manuscript includes the following:

Tables S1 to S7

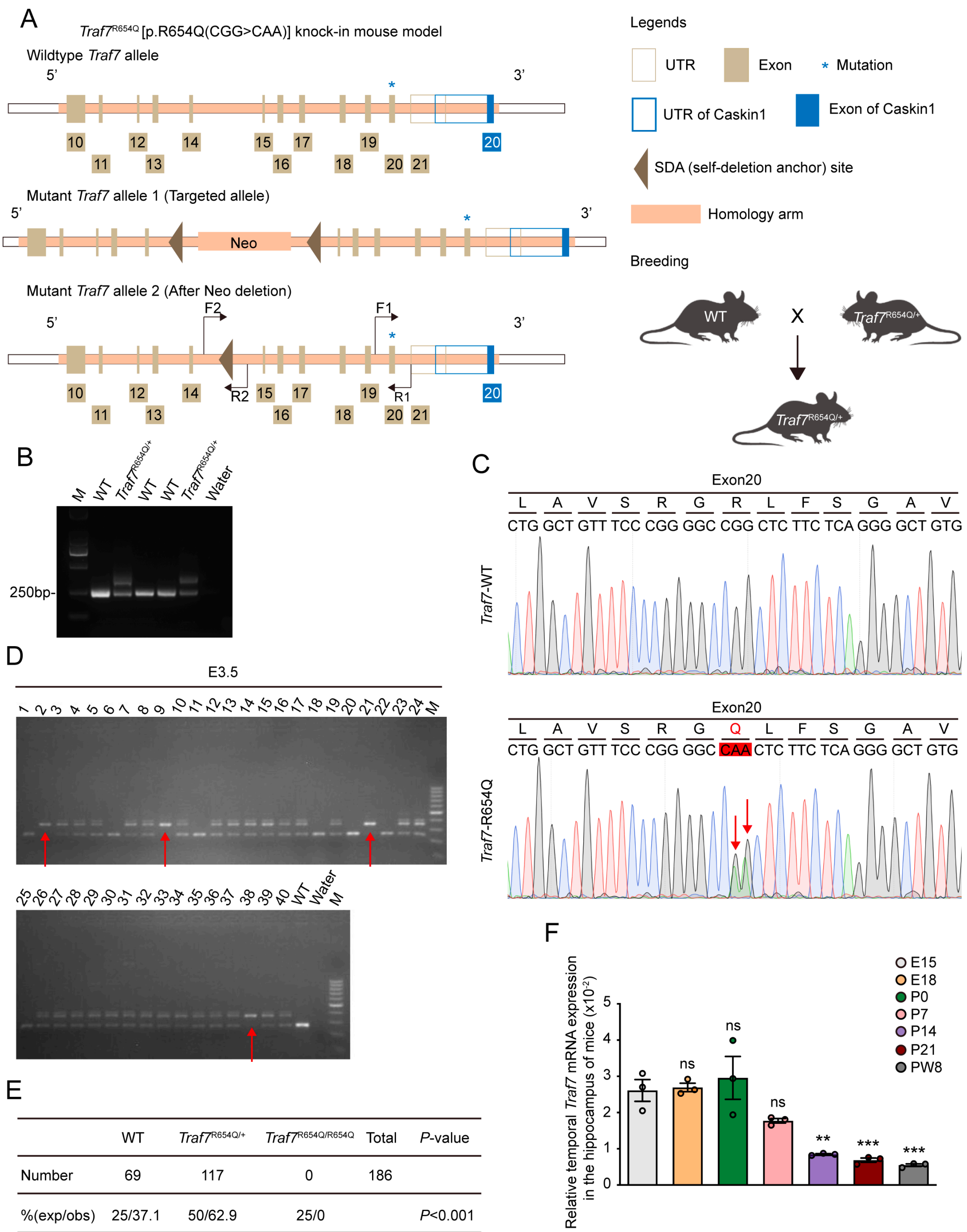

	WT	Traf7R654Q/+	Traf7R654Q/R654Q	Total	P-value
Number	69	117	0	186	
%(exp/obs)	25/37.1	50/62.9	25/0		P<0.001

Fig. S1. Homozygous *Traf7*$^{R654Q/R654Q}$ leads to embryonic mortality (related to Figure 1). (**A**) Schematic representation of *Traf7*^{R654Q}-KI mouse model generated by ES cell targeting. (**B**) PCR genotyping of KI mouse offspring. (**C**) Sanger sequencing map represents that *Traf7* gene occurred R654Q mutation. (**D**) Genotyping of forty blastocysts generated by IVF at E3.5. *Traf7*$^{R654Q/R654Q}$ blastocysts are indicated by red arrows. (**E**) Genotype distribution in litters from *Traf7*$^{R654Q/+}$ intercrosses at P9. (**F**)

Temporal expression of *Traf7* mRNA in the murine HP from E15 to PW8, measured by RT-qPCR. Data are presented as mean ± SEM. One-way ANOVA tests were used in (F). $^{**}P<0.01$, $^{***}P<0.001$; ns, not significant.

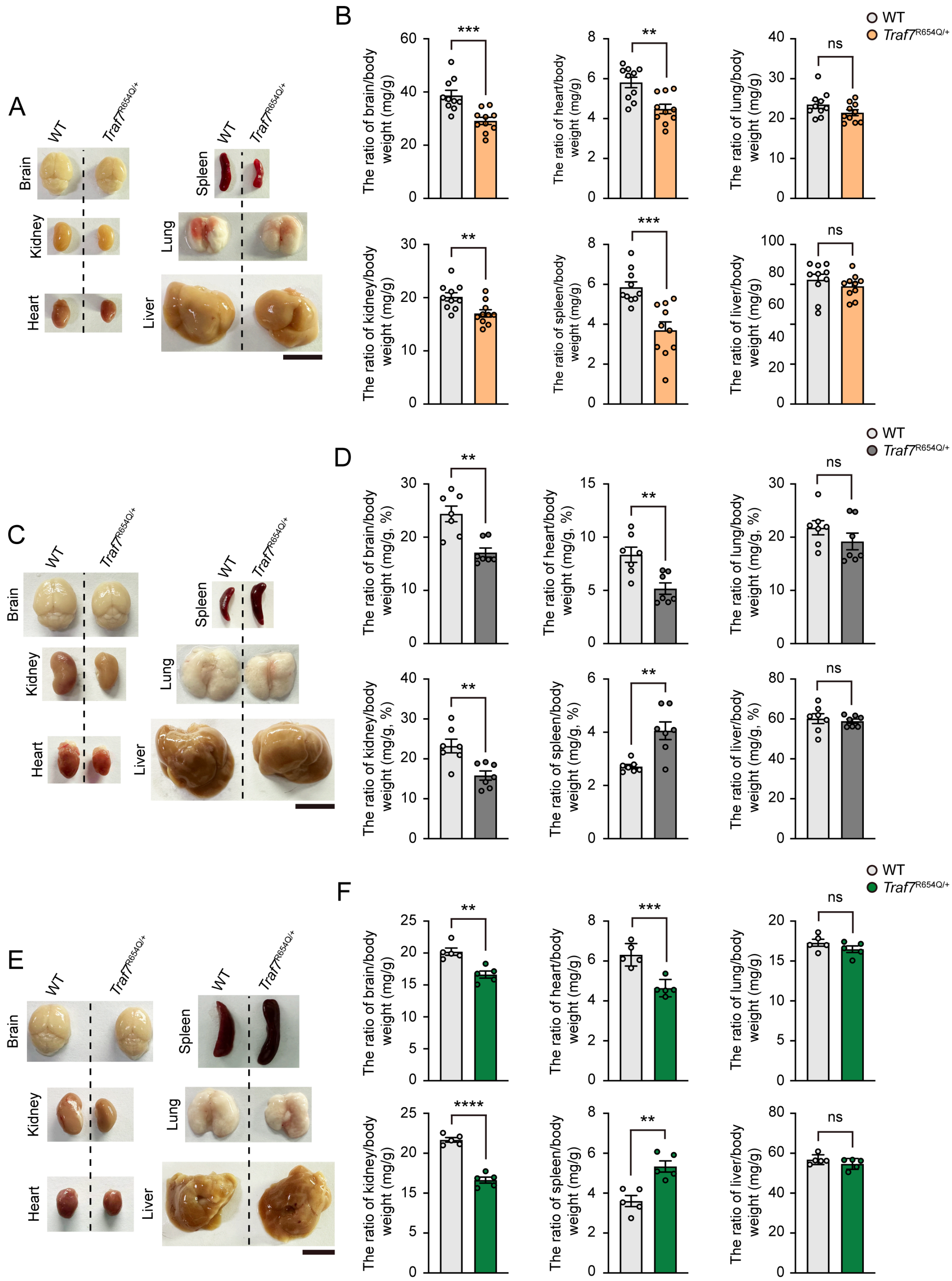

Fig. S2. Multiple organs exhibit developmental abnormalities in *Traf7*$^{R654Q/+}$ mice (related to Figure 1). (**A** and **B**) Morphology of the brain, heart, kidneys, spleen, lungs, and liver isolated at PW3 from *Traf7*$^{R654Q/+}$ mice and WT littermates (n=10). Scale bar, 1 cm. The ratio of organ weight (in milligrams) to body weight (in grams) for both groups is shown in (B). (**C** and **D**) Morphology of the brain, heart, kidneys, spleen,

lungs, and liver isolated at PW8 from *Traf7*$^{R654Q/+}$ mice and WT littermates (n=7). Scale bar, 1 cm. The ratio of organ weight (in milligrams) to body weight (in grams) for both groups is shown in (D). (**E** and **F**) Morphology of the brain, heart, kidneys, spleen, lungs, and liver isolated at PW15 from *Traf7*$^{R654Q/+}$ mice and WT littermates (n=5). Scale bar, 1 cm. The ratio of organ weight (in milligrams) to body weight (in grams) for both groups is shown in (F). Data are presented as mean ± SEM. Unpaired two-tailed Student's t-tests or Mann-Whitney U tests were used as appropriate in (B), (D), and (F). $^{**}P<0.01$, $^{***}P<0.001$, $^{****}P<0.0001$; ns, not significant.

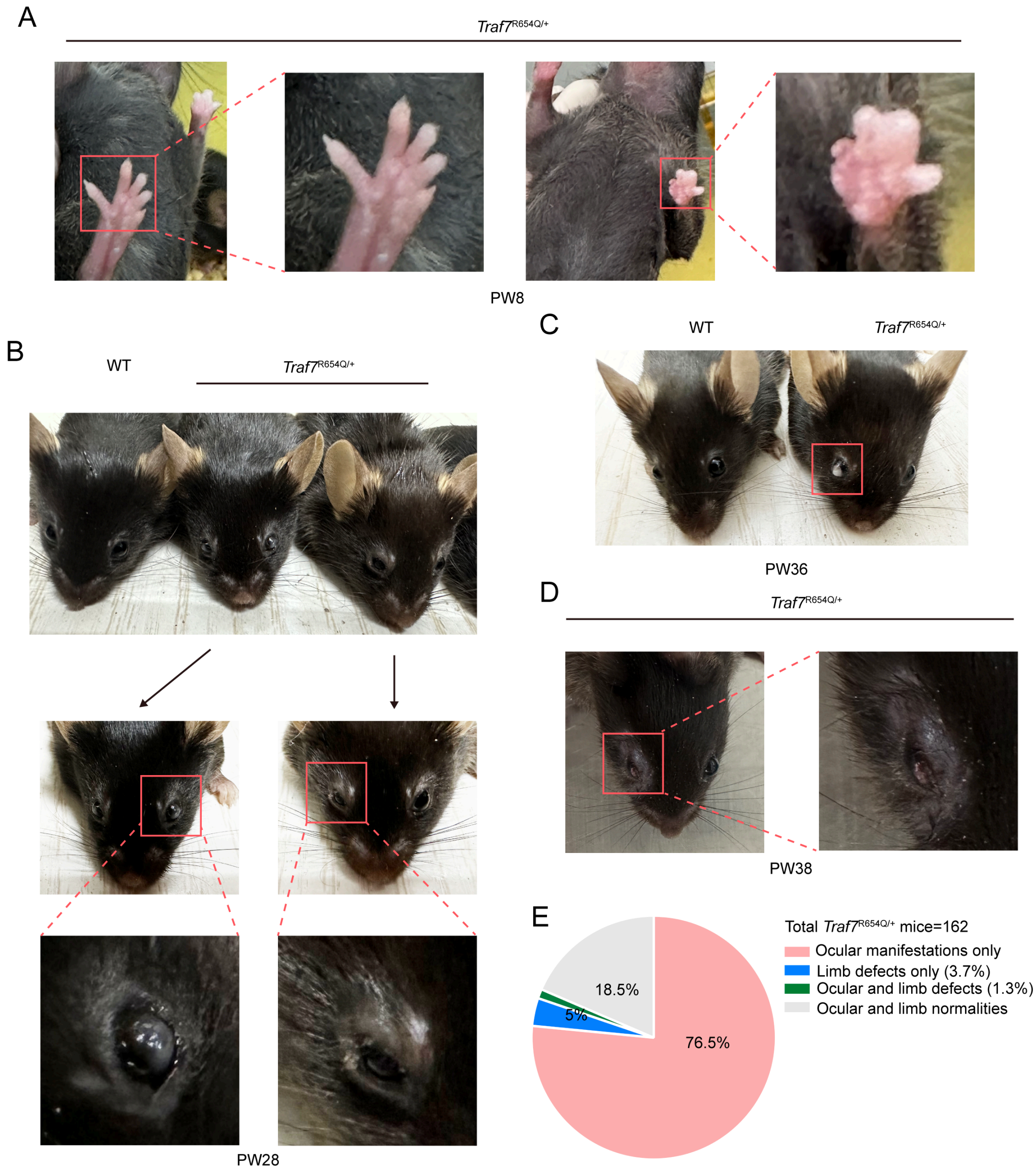

Fig. S3. *Traf7*$^{R654Q/+}$ mice exhibit ocular and limb defects (related to Figure 1). (A) *Traf7*$^{R654Q/+}$ mice displayed limbs defects compared to WT mice at PW8. WT, left; *Traf7*$^{R654Q/+}$, right. **(B-D)** *Traf7*$^{R654Q/+}$ mice exhibited ocular abnormalities at PW28, PW36, and PW38. WT, left; *Traf7*$^{R654Q/+}$, right. **(E)** Phenotype distribution of ocular and/or limb defects in *Traf7*$^{R654Q/+}$ mice at PW8. The pie chart shows the proportions of *Traf7*$^{R654Q/+}$ mice (n=162) exhibiting ocular manifestations only, limb defects only, both ocular and limb defects, or normal eyes and limbs.

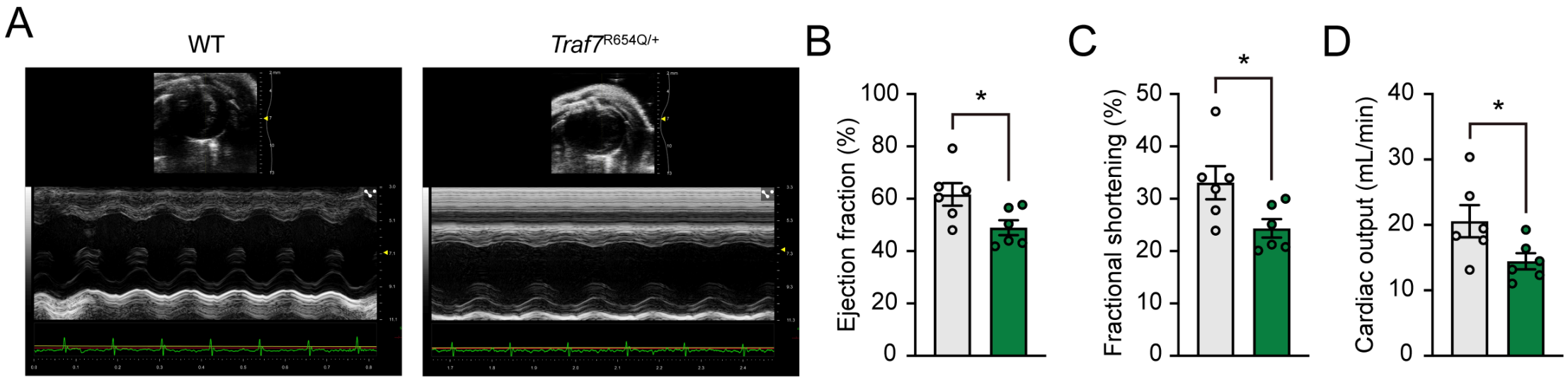

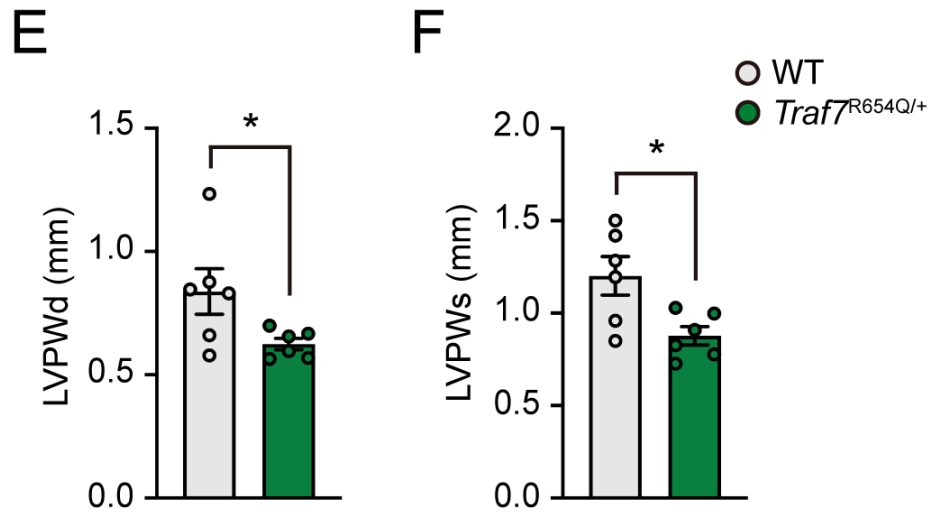

G

Features	WT (n=6)	*Traf7*R654Q/+ (n=6)	*P*-value
LVIDd (mm)	3.748	3.590	ns
LVIDs (mm)	2.533	2.733	ns
IVSd	1.811	2.272	ns
IVSs	2.274	2.620	ns
LVAWd (mm)	1.627	2.211	ns
LVAWs (mm)	2.179	2.571	ns
LV mass	201.642	259.423	ns
Volumed	65.515	59.243	ns
Volumes	26.177	30.204	ns
Heart rate (bpm)	543.9	502.8	ns

Fig. S4. Cardiac defects in *Traf7*$^{R654Q/+}$ mice (related to Figure 1). (A) Echocardiography imaging of cross-sectional cardiac plane from *Traf7*$^{R654Q/+}$ and WT mice (n=6) at PW8. (**B-G**) Summary of cross-sectional cardiac echocardiography data from *Traf7*$^{R654Q/+}$ and WT mice (n=6) at PW8. Cardiac function parameters: EF, FS, cardiac output, LVPWd/s, LVAWd/s, LVIDd/s, and heart rate. Data are presented as mean ± SEM. Unpaired two-tailed Student's t-tests were used in (B-G). *$P<0.05$; ns, not significant.

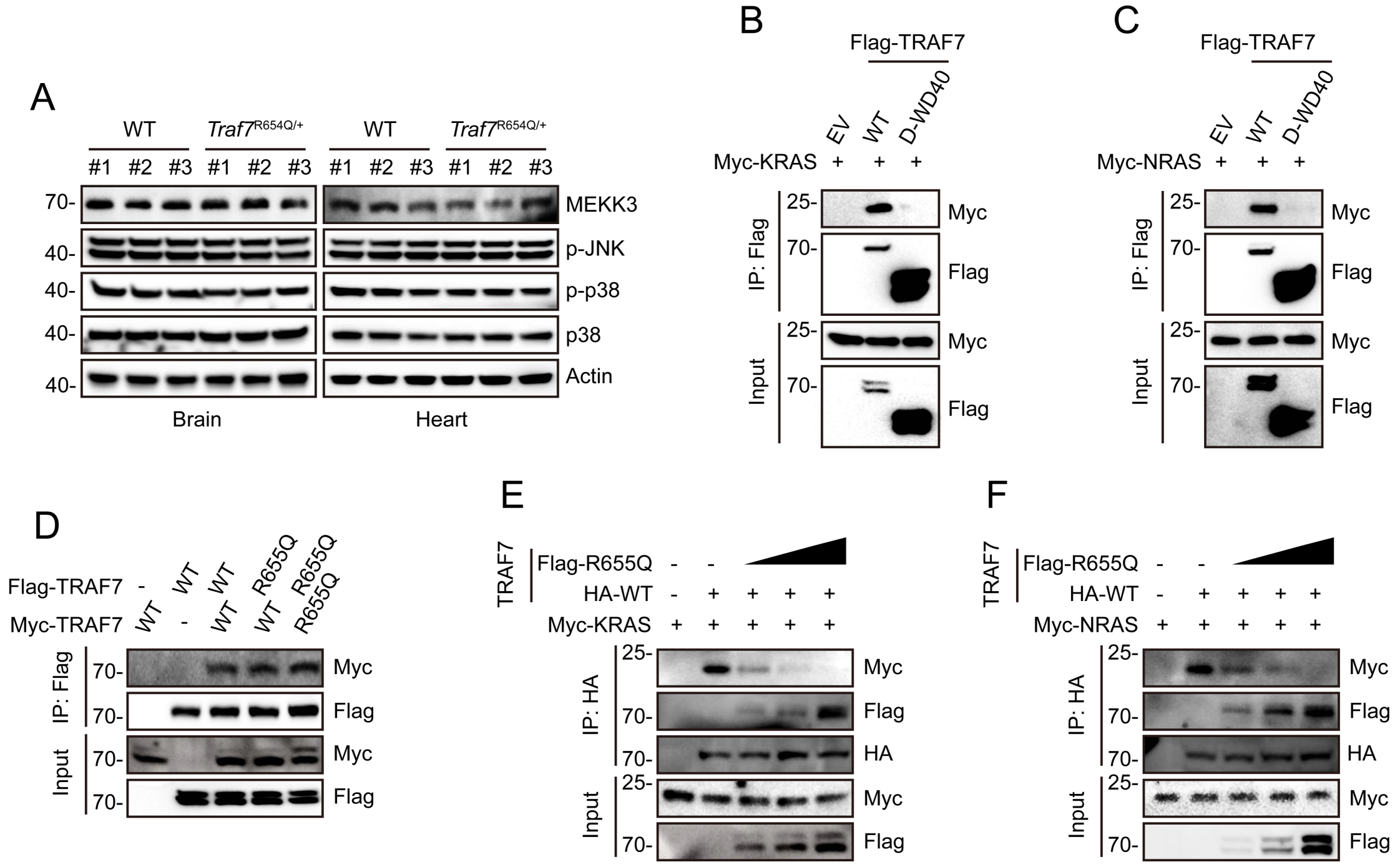

Fig. S5. CAFDADD-associated TRAF7 mutants exert dominant-negative effects on the interaction between TRAF7-WT and KRAS/NRAS (related to Figure 3 and Figure 4). (**A**) WB analysis of indicated proteins in WCLs from brain and heart tissues of *Traf7*$^{R654Q/+}$ and WT mice. (**B** and **C**) The WCLs from HEK293T cells transfected with indicated plasmids were prepared and subjected to co-IP with anti-Flag antibody, followed by WB analysis. Flag-TRAF7-D-WD40 denotes the WDR domain deletion mutant. (**D**) Co-IP assay showing that TRAF7-WT and -R655Q mutant form homo- and hetero-dimers. (**E** and **F**) HEK293T cells were transfected with indicated plasmids which included TRAF7-R655Q (1, 2, 4 μg) and TRAF7-WT (4 μg). The WCLs were prepared and subjected to co-IP with anti-HA antibody, followed by WB analysis.

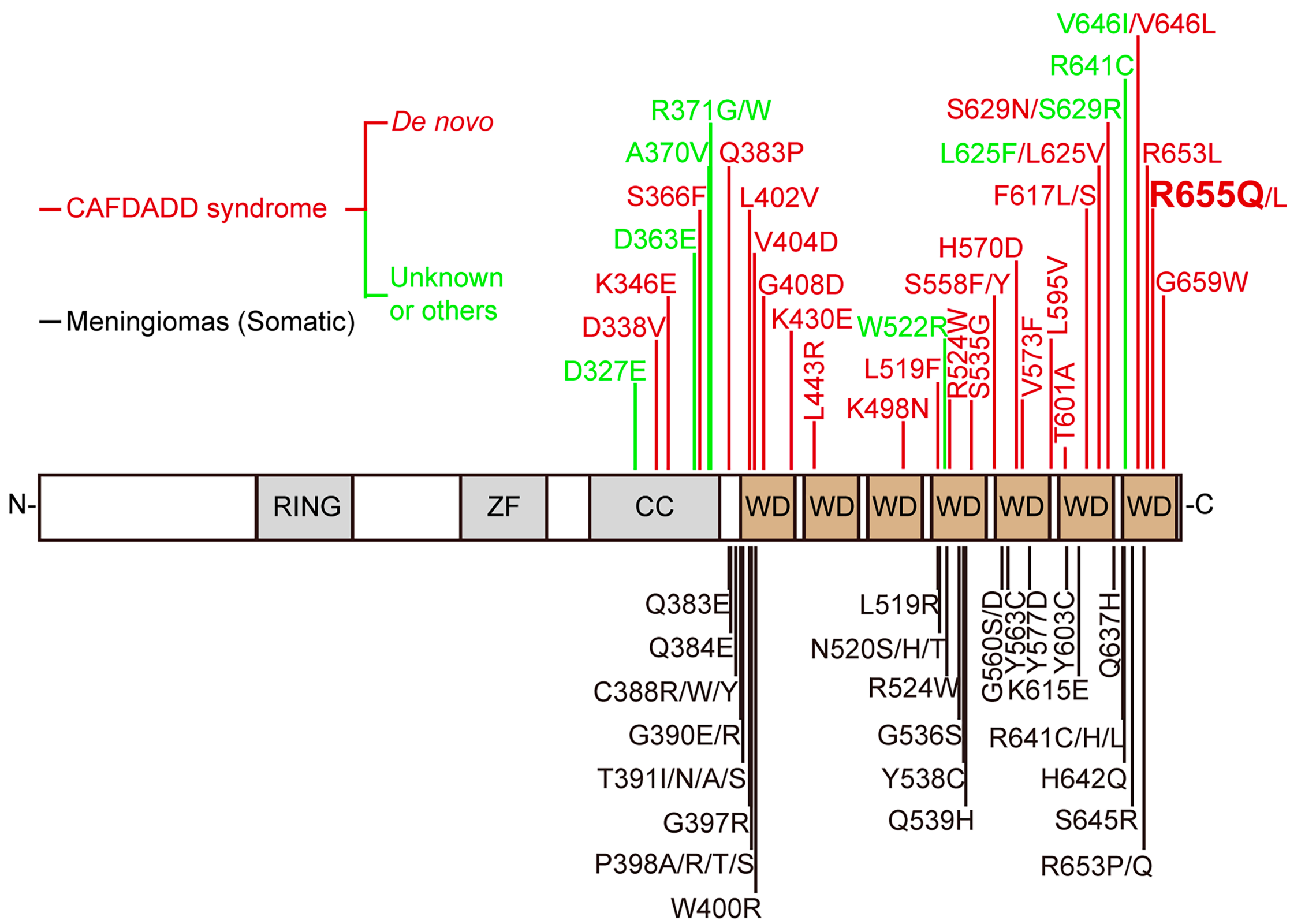

Fig. S6. *TRAF7* variants in diseases (related to Figure 4). Domain boundaries are drawn approximately to scale, based on Ref (*5*). CAFDADD-associated *TRAF7* variants are indicated in green (mutations with unknown significance, inheritance, mosaic, or others) or red (*de novo* mutations). In black, those reported in meningiomas.

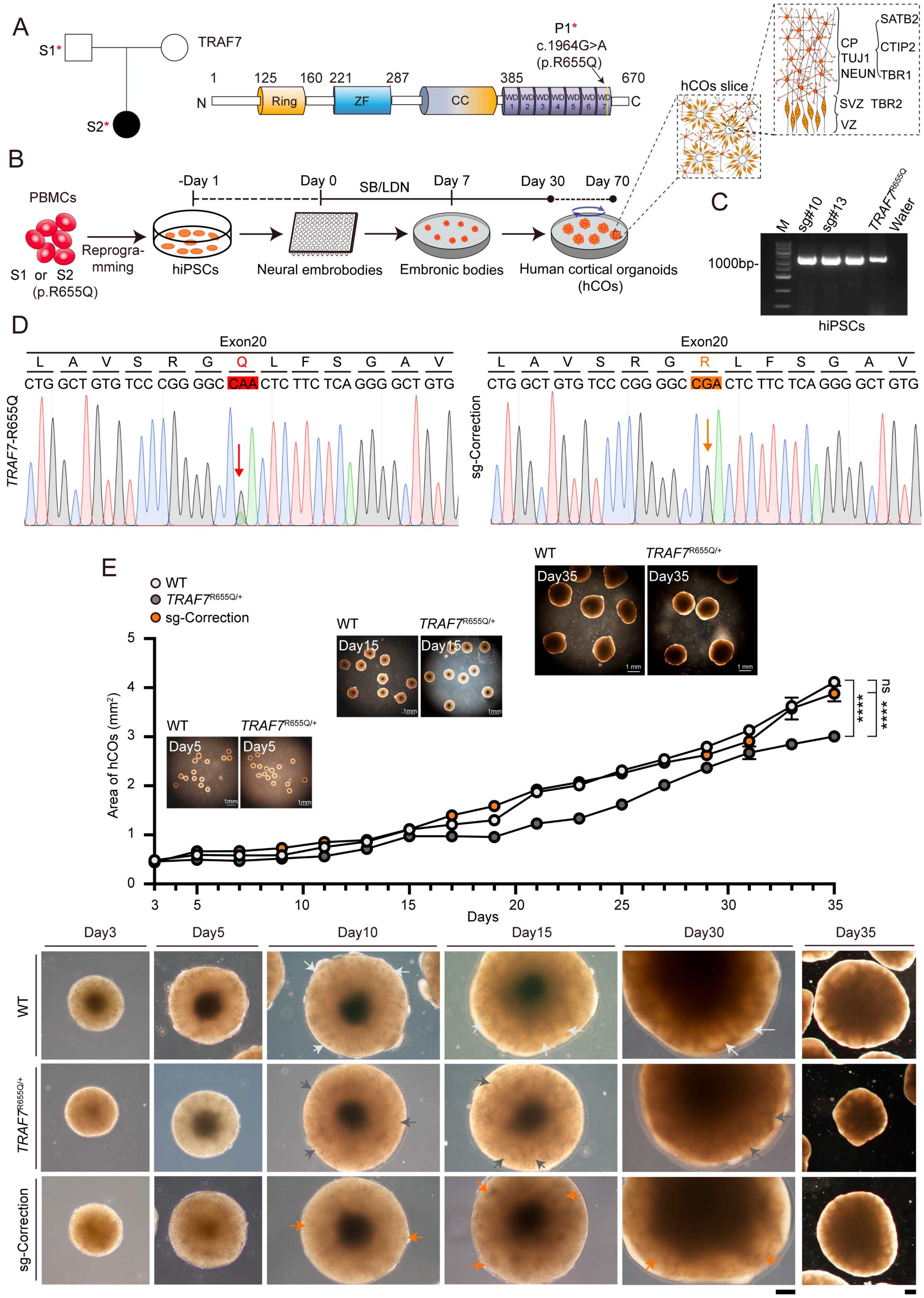

Fig. S7. ***TRAF7*$^{R655Q/+}$ hCOs show reduced size (related to Figure 5).** (**A**) Pedigree and schematic view of the TRAF7 protein domains. Square, male; circle, female; filled symbol, affected. S1 and S2 (red asterisks) denote the cell lines used in this study. (**B**) Schematic of the experimental workflow for hCOs generation. (**C**) PCR genotyping of

iPSCs clones. (**D**) Sanger sequencing confirming the correction of the *TRAF7*^{R655Q} mutation in the isogenic control line. (**E**) Representative bright-field images of hCOs (n=20) during culture and their growth curves throughout differentiation. Arrows indicate neural tubes. Scale bar, 200 μm (left), 500 μm (right). Data are presented as mean ± SEM. Two-way ANOVA tests were used in (E). ****P<0.0001; ns, not significant.

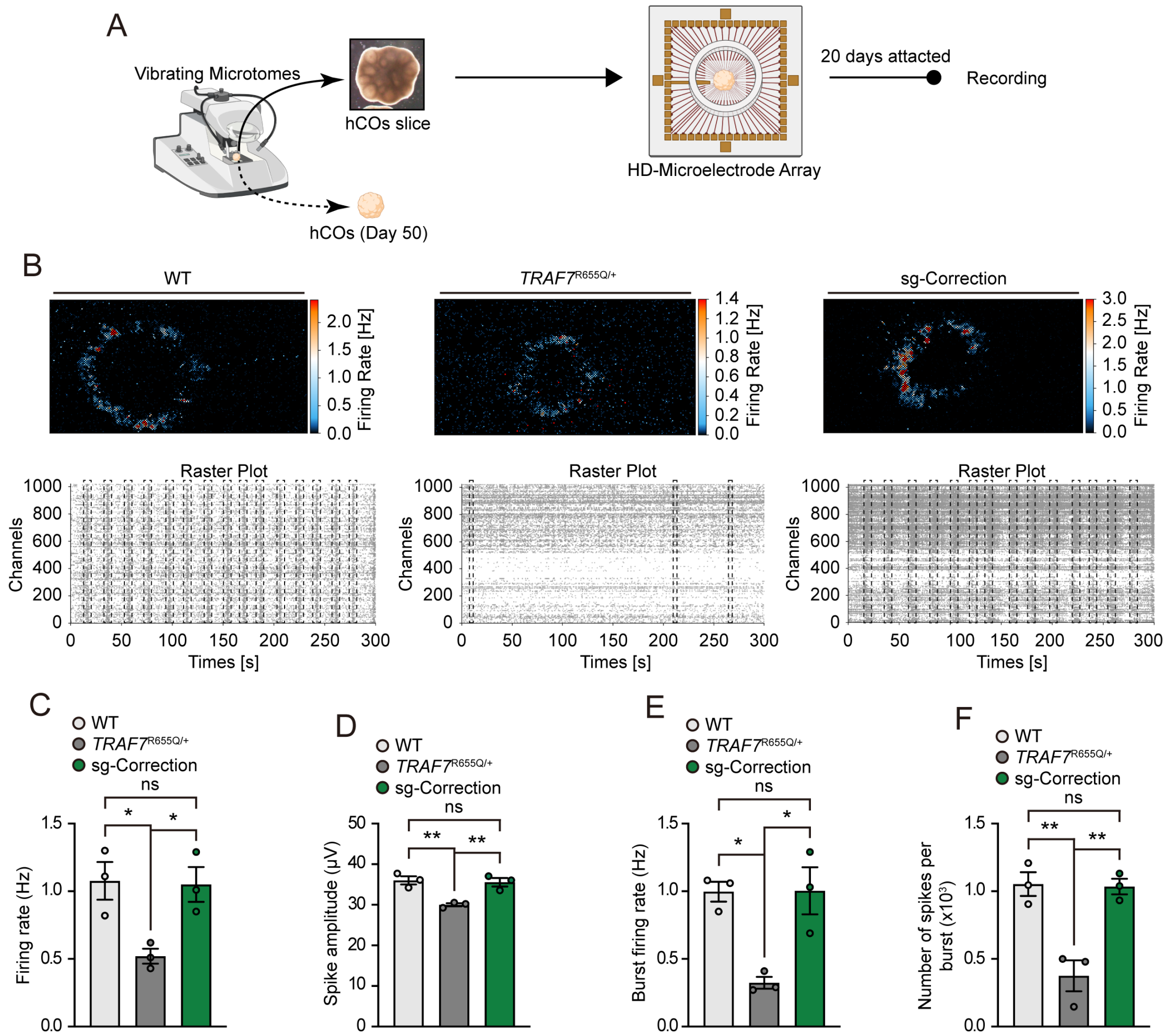

Fig. S8. CAFDADD patient-derived *TRAF7*$^{R655Q/+}$ hCOs exhibit neuronal functional deficits (related to Figure 5). (**A**) Schematic of the MEA assay in this study. (**B**) Magnified view of representative neuronal activity of hCOs. (**C**-**F**) Quantification of electrophysiological properties: spike firing rate (C), spike amplitude (D), burst peak firing rate (E), and number of spikes per burst (F). Data are presented as mean ± SEM. One-way ANOVA tests were used in (C-F). **P*<0.05, ***P*<0.01; ns, not significant.

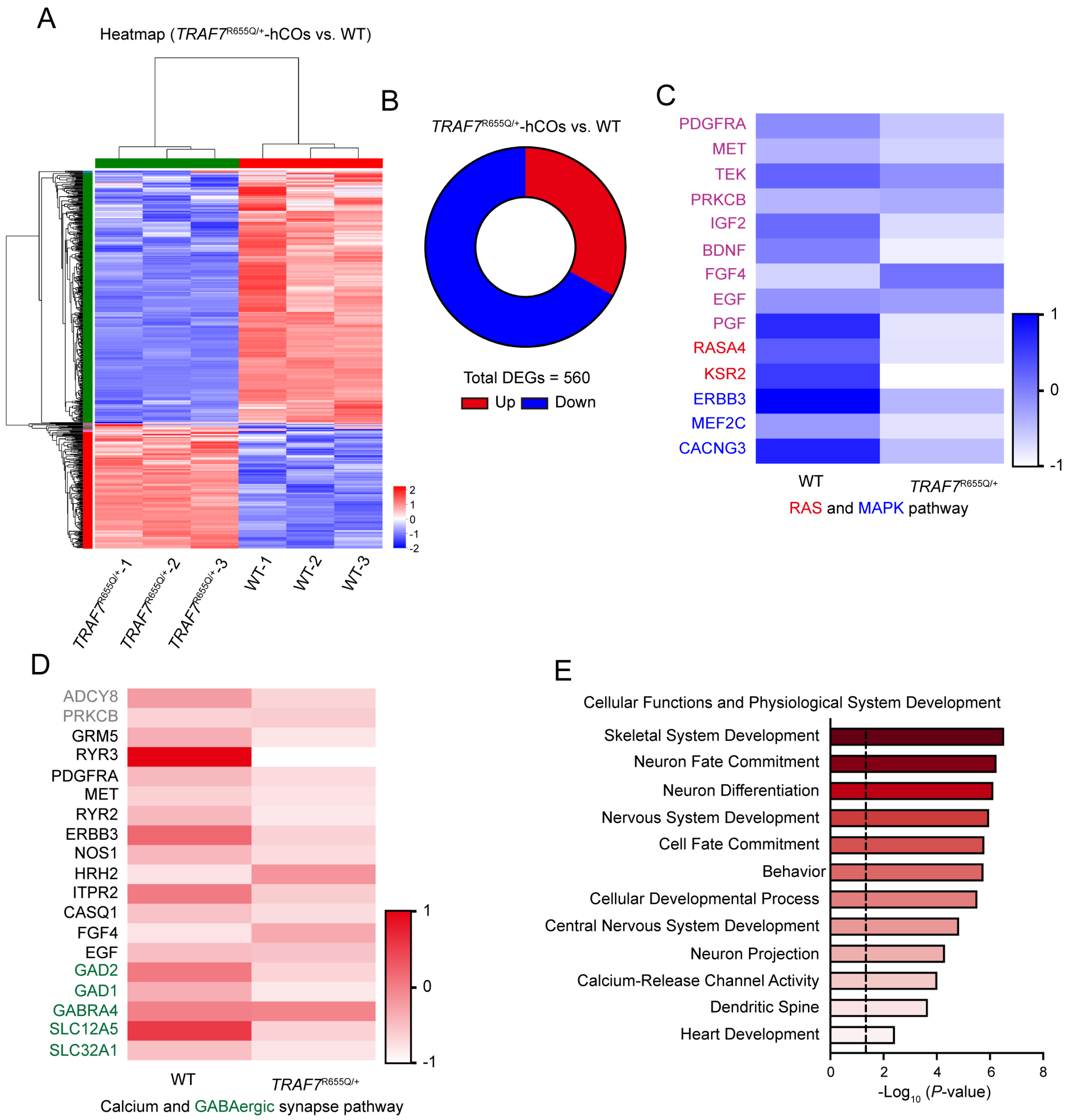

Fig. S9. Transcriptome analysis of hCOs bearing the *TRAF7*^{R655Q} mutation (related to Figure 7). (**A**) Hierarchical clustering and heatmap illustration of DEGs from RNA-Seq analysis in *TRAF7*$^{R655Q/+}$ and WT hCOs. Color key denotes Z-score of normalized expression. (**B**) Proportions of up- and down-regulated transcripts in *TRAF7*$^{R655Q/+}$ and WT hCOs. (**C**) KEGG pathway analysis shows enrichment of DEGs in RAS and MAPK signaling pathways. Color scale from white (lowest) to blue (highest). DEGs are highlighted as follows: purple, RAS-MAPK pathway; red, RAS pathway only; blue, MAPK pathway only. (**D**) KEGG pathway analysis shows enrichment of DEGs in calcium and GABAergic synapse pathways. Color scale from white (lowest) to red (highest). DEGs are highlighted as follows: gray, both pathways;

black, calcium pathway only; green, GABAergic synapse only. (**E**) GO analysis shows selected overrepresented categories of cellular functions and physiological system development. The number of DEGs associated with each category is specified. A $-\text{Log}_{10}$ (*P*-value)>1.3 was considered significant.

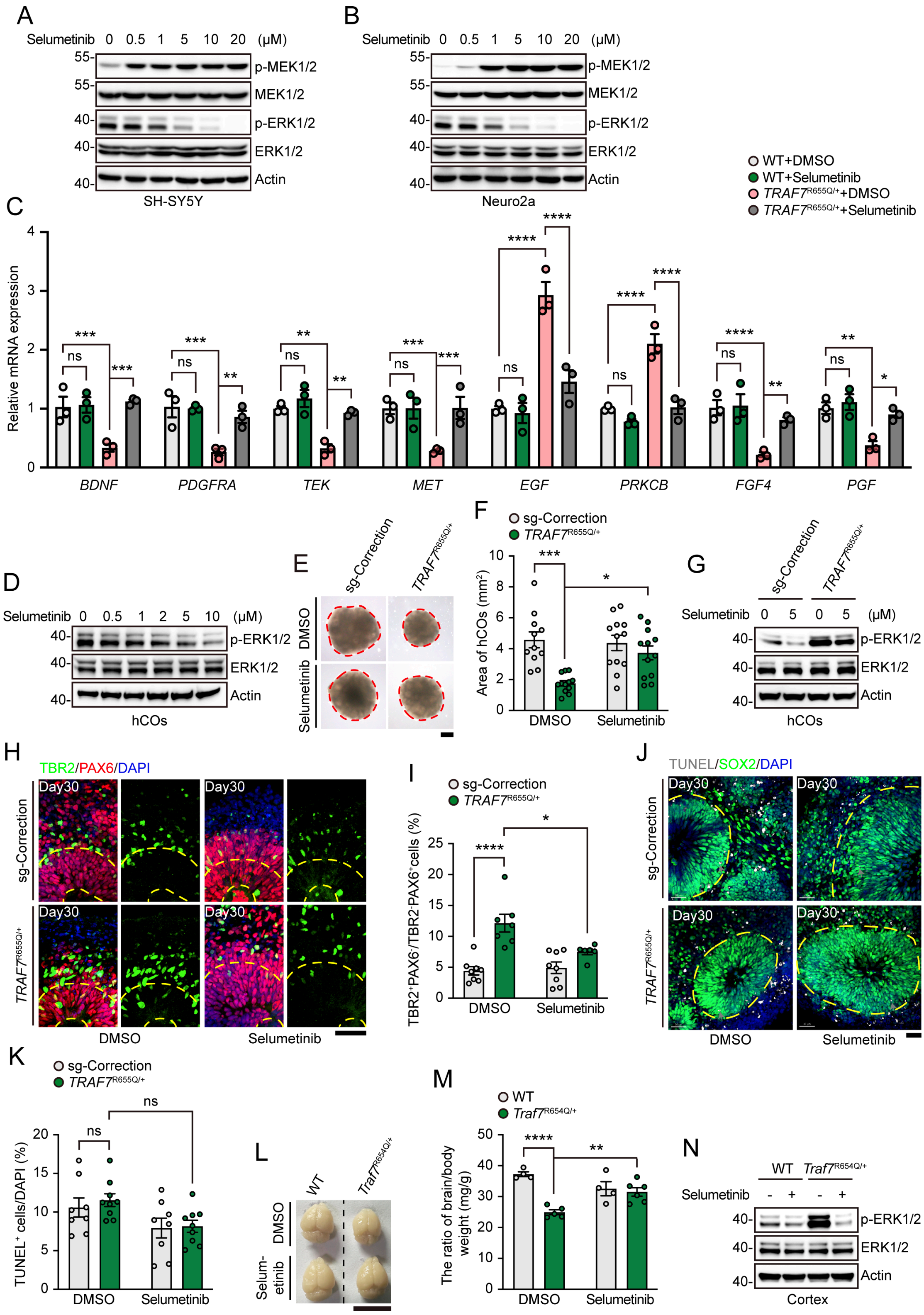

A
Selumetinib 0 0.5 1 5 10 20 (μM)
p-MEK1/2
MEK1/2
p-ERK1/2
ERK1/2
Actin
SH-SY5Y
B
Neuro2a
C
WT+DMSO
WT+Selumetinib
TRAF7R655Q/+ +DMSO
TRAF7R655Q/+ +Selumetinib
Relative mRNA expression
BDNF
PDGFRA
TEK
MET
EGF
PRKCB
FGF4
PGF
D
Selumetinib 0 0.5 1 2 5 10 (μM)
hCOs
E
sg-Correction
DMSO
Selumetinib
F
Area of hCOs (mm²)
G
H
TBR2/PAX6/DAPI
Day30
I
TBR2+PAX6-/TBR2-PAX6+cells (%)
J
TUNEL/SOX2/DAPI
K
TUNEL+ cells/DAPI (%)
L
WT
M
The ratio of brain/body weight (mg/g)
N
Cortex

analysis of indicated proteins in WCLs from SH-SY5Y and Neuro2a cells treated with increasing concentrations of selumetinib for 48 hours. (**C**) Selumetinib partially restores the expression of indicated RAS-MAPK pathway-related genes in *TRAF7*$^{R655Q/+}$ hCOs, measured by RT-qPCR. (**D**) WB analysis of indicated proteins in WCLs from hCOs treated with increasing concentrations of selumetinib below 10 μM for 24 hours. (**E** and **F**) Representative bright-field images and quantification of hCOs morphology (n=6) following treatment with selumetinib. (**G**) WB analysis of indicated proteins in WCLs from hCOs following selumetinib treatment. (**H** and **I**) Representative images and quantification of TBR2^{+}PAX6^{-} cells in *TRAF7*$^{R655Q/+}$ hCOs (n=6) after selumetinib treatment. The circles in the statistical graph represent the number of fields that were counted. Scale bar, 30 μm. (**J** and **K**) Representative images and quantification of apoptosis in hCOs (n=6) after selumetinib treatment. The circles in the statistical graph represent the number of fields that were counted. TUNEL^{+} cells are indicated with gray arrows. Scale bar, 30 μm. (**L** and **M**) Morphology of brain isolated at P21 from *Traf7*$^{R654Q/+}$ mice treated with selumetinib (n=6) or vehicle (n=5), and WT littermates treated with selumetinib (n=4) or vehicle (n=4). Scale bar, 1 cm. Quantification of the ratio of brain weight (in milligrams) to body weight (in grams) is shown in (M). (**N**) WB analysis of indicated proteins in cortical WCLs from *Traf7*$^{R654Q/+}$ and WT mice following selumetinib treatment. Data are presented as mean ± SEM. Two-way ANOVA tests were used in (C), (F), (I), (K), and (M). *P<0.05, **P<0.01, ***P<0.001, ****P<0.0001; ns: not significant.

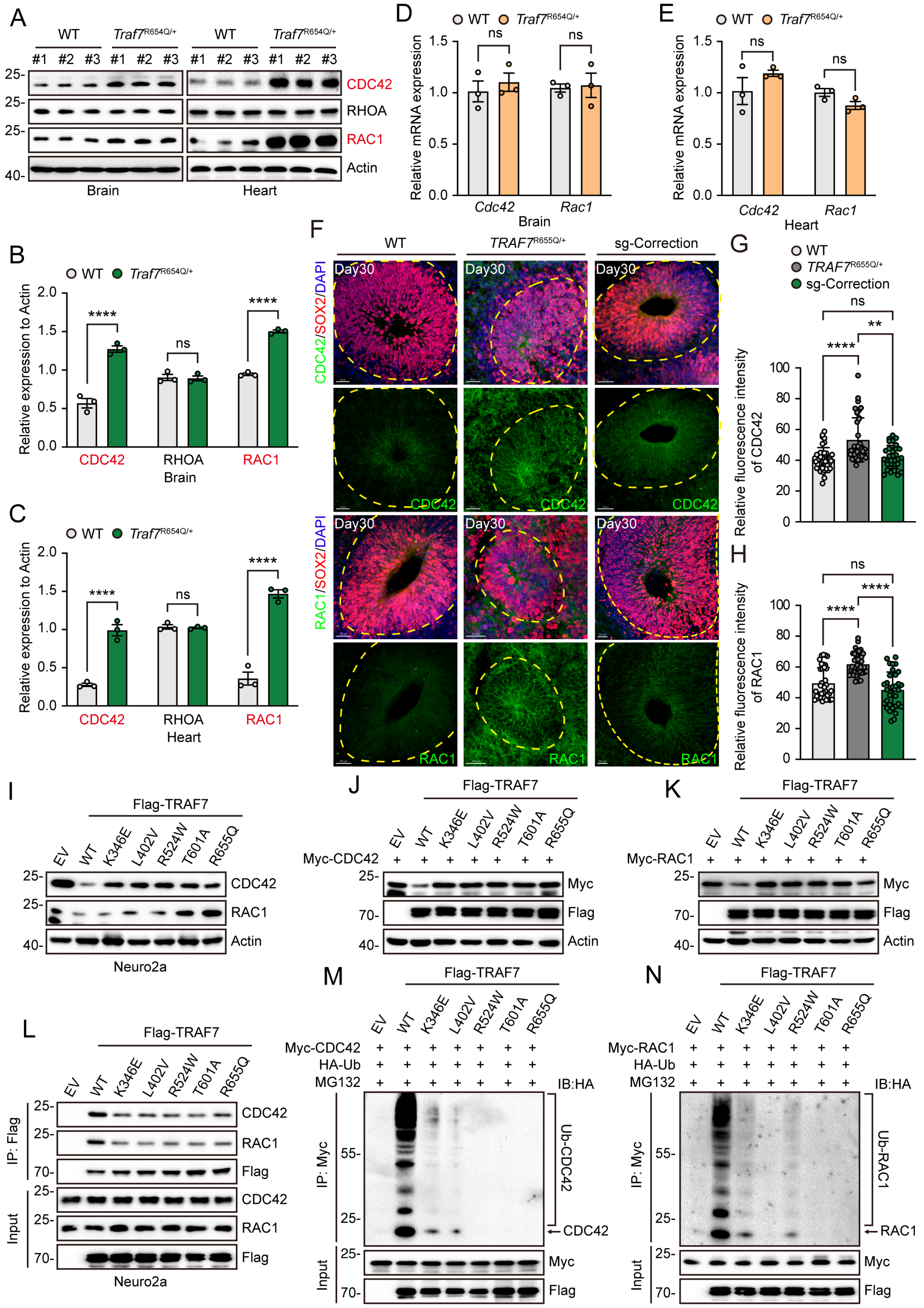

Fig. S11. CAFDADD-associated *TRAF7* mutants are defective in promoting ubiquitination and degradation of CDC42/RAC1. (**A**) WB analysis of indicated proteins in WCLs from brain and heart tissues of *Traf7*$^{R654Q/+}$ and WT mice. (**B and C**) Quantification of indicated proteins from (A), normalized to the intensity of Actin. (**D**

and **E**) *Cdc42* and *Rac1* mRNA expression in brain (D) or heart (E) tissues of *Traf7*$^{R654Q/+}$ and WT mice, measured by RT-qPCR. (**F**-**H**) Comparison of the intensity of CDC42 (upper panels) or RAC1 (lower panels) staining in the hCOs (n=5). The circles in the statistical graph represent the number of hCO slices that were counted. Scale bar, 30 μm. (**I**) WB analysis of WCLs from Neuro2a cells transfected with indicated plasmids. (**J** and **K**) WB analysis of WCLs from HEK293T cells transfected with indicated plasmids. (**L**) Neuro2a cells were transfected with indicated plasmids. The WCLs were prepared and subjected to co-IP with anti-Flag antibody, followed by WB with indicated antibodies. (**M** and **N**) *In vivo* ubiquitination assays in HEK293T cells transfected with indicated plasmids, analyzed by WB. Data are presented as mean ± SEM. Two-way ANOVA tests were used in (B-E). Kruskal-Wallis H tests were used in (G) and (H). $^{**}P<0.01$, $^{****}P<0.0001$; ns, not significant.

Other Supplementary Material for this manuscript includes the following:

Tables S1 to S7

Supplementary Table S1: Genotype and phenotype of CAFDADD cases carrying germline *TRAF7* mutations from published studies.

Supplementary Table S2: Detailed list of DEGs (*P*adjust value<0.05 and |Log2 fold change|≥1) identified in *TRAF7*$^{R655Q/+}$-patient hCOs by RNA-Seq.

Supplementary Table S3: Enriched signaling and metabolic canonical pathways identified in *TRAF7*$^{R655Q/+}$-patient hCOs by RNA-Seq.

Supplementary Table S4: Enriched cellular functions and physiological system development identified in *TRAF7*$^{R655Q/+}$-patient hCOs by RNA-Seq.

Supplementary Table S5: Sequence information.

Supplementary Table S6: Cell cultures, materials, and chemicals.

Supplementary Table S7: Antibody information.
